# A multiscale modelling approach to assess the impact of metabolic zonation and microperfusion on the hepatic carbohydrate metabolism

**DOI:** 10.1371/journal.pcbi.1006005

**Published:** 2018-02-15

**Authors:** Nikolaus Berndt, Marius Stefan Horger, Sascha Bulik, Hermann-Georg Holzhütter

**Affiliations:** 1 Computational Biochemistry Group, Institute of Biochemistry, Charite—University Medicine Berlin, Charitéplatz 1, Berlin; 2 Department of Diagnostic and Interventional Radiology, Eberhard-Karls-University Tubingen, Tuebingen, Germany; 3 German Federal Institute for Risk Assessment, Junior Research Group Supply-Chain-Models, Max-Dohrn-Straße 8–10, Berlin, Germany; University of Michigan, UNITED STATES

## Abstract

The capacity of the liver to convert the metabolic input received from the incoming portal and arterial blood into the metabolic output of the outgoing venous blood has three major determinants: The intra-hepatic blood flow, the transport of metabolites between blood vessels (sinusoids) and hepatocytes and the metabolic capacity of hepatocytes. These determinants are not constant across the organ: Even in the normal organ, but much more pronounced in the fibrotic and cirrhotic liver, regional variability of the capillary blood pressure, tissue architecture and the expression level of metabolic enzymes (zonation) have been reported. Understanding how this variability may affect the regional metabolic capacity of the liver is important for the interpretation of functional liver tests and planning of pharmacological and surgical interventions. Here we present a mathematical model of the sinusoidal tissue unit (STU) that is composed of a single sinusoid surrounded by the space of Disse and a monolayer of hepatocytes. The total metabolic output of the liver (arterio-venous glucose difference) is obtained by integration across the metabolic output of a representative number of STUs. Application of the model to the hepatic glucose metabolism provided the following insights: (i) At portal glucose concentrations between 6–8 mM, an intra-sinusoidal glucose cycle may occur which is constituted by glucose producing periportal hepatocytes and glucose consuming pericentral hepatocytes, (ii) Regional variability of hepatic blood flow is higher than the corresponding regional variability of the metabolic output, (iii) a spatially resolved metabolic functiogram of the liver is constructed. Variations of tissue parameters are equally important as variations of enzyme activities for the control of the arterio-venous glucose difference.

## Author summary

Glucose homeostasis is one of the central liver functions. The liver extracts glucose from the blood when plasma glucose levels are high and produces glucose when plasma glucose levels are low. To fulfill this function the liver is organized in smallest functional units, the sinusoidal tissue units (STUs). These STUs consist of a single sinusoid surrounded by linear arranged hepatocytes. Liver zonation describes the spatial separation of metabolic pathways along the STUs. As blood flows through the sinusoid the plasma nutrient and hormone composition changes and in conjunction with the heterogeneous endowment of metabolic enzymes this leads to big differences in the metabolic performance of hepatocytes depending on their position within the sinusoid. This makes liver zonation and blood flow two central determinants for the functional output of the liver. In this work we present a tissue model of hepatic carbohydrate metabolism that combines liver zonation and microperfusion within the STU. We show that structural properties, enzymatic properties and regional bloodflow are equally important for the understanding of liver functionality. With our work we provide a true multi-scale model bridging the scale from the cellular to the tissue level.

## Introduction

In mammals, the liver is the central organ for the control of plasma glucose. To restrict variations of plasma glucose to the range between 3 mM (after exercise or moderate fast [1]) and 10 mM (after meal ingestion, [2]), liver metabolism may switch between glucose production (gluconeogenesis and glycogenolysis) and glucose consumption. At the cellular level, this switch is accomplished by the interplay of multiple regulatory mechanisms including long term alterations in the abundance of metabolic enzymes, alterations in kinetic properties of key regulatory enzymes due to external hormonal regulation, alterations in enzymatic activity in response to internal substrate availability and allosteric regulation.

The majority of metabolic functions of the liver are confined to hepatocytes. These cells form tightly connected cell layers that are separated from the liver capillaries (sinusoids) by the space of Disse [3]. The transport of plasma metabolites from the lumen of sinusoids through the space of Disse into the cytosol of hepatocytes and vice versa connects the cellular metabolism with the blood plasma. Thus, blood flow is a crucial determinant of the liver's overall metabolic output [4].

The importance to include blood perfusion and the tissue architecture into a quantitative estimate of liver metabolism has been stressed in an earlier modeling work of Chalhoub und Belovich [5]. However, this simple truth is routinely neglected in experimental and modeling studies where the metabolic capacity of the hepatocyte (assessed in cell cultures) is wrongly equated with the metabolic capacity of the organ.

To better understand the role of blood perfusion and tissue structure for the metabolic performance of the liver, we have developed a multi-scale tissue model of the so-called sinusoidal tissue unit (STU). We define a single STU by an ensemble of a single sinusoid, the accompanying space of Disse and the adjacent layers of hepatocytes. The sinusoids form the microvascular bed linking the hepatic portal vein and artery with the hepatic central vein. The exchange of substances between the blood, the space of Disse and the hepatocytes leads to a progressive alteration of plasma composition along the STU, continuously changing from the portal region (where the blood from the liver artery and portal vein mix) to the central region connected to the liver vein.

Importantly, hepatocytes at different spatial positions of the STU display a differential endowment with metabolic enzymes. Higher amounts of the glycolytic enzymes glucokinase (GK), PFK2/FBP2, phosphofructokinase 1 (PFK1), pyruvate kinase (PK) are found in the pericentral region, while higher enzyme levels of the gluconeogenetic enzymes glucose-6-phosphate phosphatase (G6PP), fructose1,6bisphosphatase (FBP1), mitochondrial pyruvate carboxylase (PCmito) and PEPCK are found in the periportal region [6–21]. Consistent with such a heterogeneous distribution of enzyme activities, the glycogen content of periportal and percentral hepatocytes may largely differ [22]. These findings point to a zonated carbohydrate metabolism in the liver: Periportal hepatocytes are more engaged in glucose production, while pericentral hepatocytes are generally more engaged in glucose utilization [9, 10].

In a previous work [23], we constructed a detailed kinetic model of liver glucose metabolism and examined the relative importance of the different modes of enzyme regulation for the dynamic behavior of hepatic glucose metabolism. However, the influence of tissue architecture and blood flow on the ability of the liver to function as glucose homeostat were not considered in this model. It is obvious that the unique sinusoidal structure allows an efficient nutrient exchange, but how alterations in this architecture impacts on liver function is largely unknown. Therefore, in this paper we combined our previously published model of the hepatocyte carbohydrate metabolism with a model of sinusoidal blood flow that comprises some important structural and functional tissue parameters as the thickness of sinusoids, width and number of endothelial fenestrae, thickness of the space of Disse, volume of hepatocytes and diffusion coefficients of organic molecules in the various compartments of the STU. We accounted for heterogeneous enzyme expression by constructing distinct metabolic models for the periportal and pericentral hepatocyte. The model was used to investigate the relative impact of metabolic zonation, tissue architecture and microperfusion on the hepatic carbohydrate metabolism.

## Methods

### Description of the mathematical model

#### Glucose metabolism of single hepatocytes

**Fig 1:**
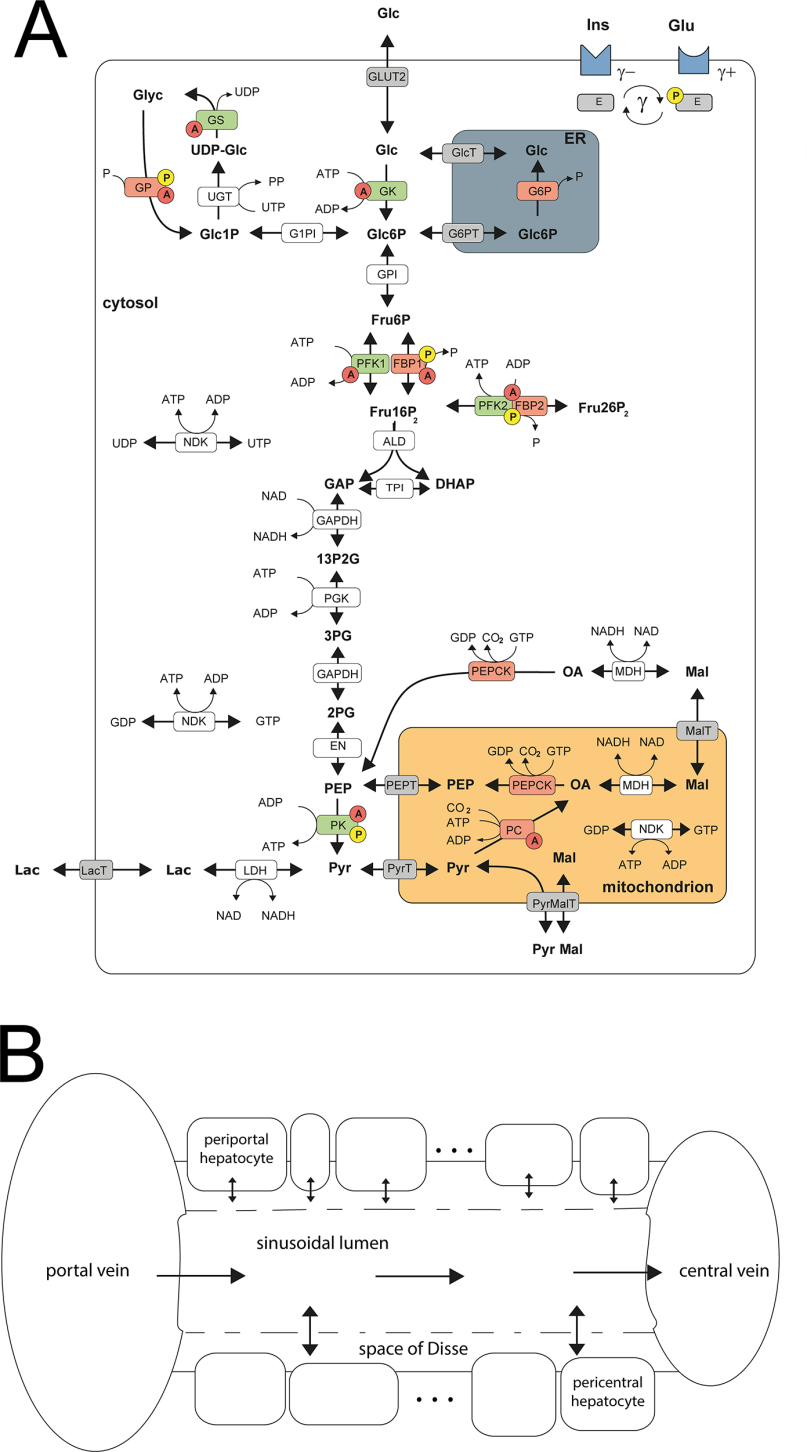
*Schematic model representation* (A) model of carbohydrate metabolism describing glycolysis, glyconeogenesis and glycogen synthesis and utilization. The model describes the enzymes Glucokinase (GK), Glucose-6-phosphate isomerase (GPI), Phosphofructokinase 1 (PFK1), Aldolase (ALD), Triosephosphate isomerase (TPI), Glyceraldehydephosphate dehydrogenase (GAPDH), Phosphoglycerate kinase (PGK), Phosphoglycerate mutase (PGM), Enolase (EN), Pyruvate kinase (PK) Lactate dehydrogenase (LDH), Glucose-6-phosphate phosphatase (G6P), Phosphofructokinase 2 (PFK2), Fructose-2,6-bisphosphatase (FBP2), Fructose-1,6-bisphosphatase (FBP1), Phosphoenolpyruvate carboxykinase (PEPCK), Pyruvate carboxylase (PC), Nucleoside-diphosphate kinase (NDK), Malate dehydrogenase (MDH), Pyrophosphatase (PPASE), Glucose-1-phosphate isomerase (P1PI), Glycuronosyltransferase (UGT), Glycogen phosphorylase (GP), Glycogen synthase (GS) and transporters (ER <-> cytosol: Glucose-6-phosphate transporter (Glc6PT), Glucose transporter (GlcT); mitochondrion <-> cytosol: Pyruvate transporter (PYRT), Phosphoenolpyruvate transporter (PEPT), Malate transporter (MALT); extern <-> cytosol: Glucose transporter 2 (GLUT2), Lactate transporter (LACT). Enzymes that are phosphorylated or dephosphorylated in response to insulin (Ins) and glucagon (Glu) stimulus are marked by a yellow P, allosteric modification of enzymes is marked by a red A. The model contains the metabolites: glucose (Glc), glucose-6-phosphate (Glc6P), fructose-6-phosphate (Fru6P), fructose-1,6-bisphosphate (Fru16P_2_), glyceraldehydephosphate (GraP), dihydroxyacetonephosphate (DHAP), 1,3-bisphosphoglycerate (13P2G), 3-phosphoglycerate (3PG), 2-phosphoglycerate (2PG), phosphoenolpyruvate (PEP), pyruvate (Pyr), lactate (Lac), malate (Mal), oxaloacetate (OA), glucose-1-phosphate (Glc1P), UDP-glucose (UDP-glc), glycogen, fructose-2,6-bisphosphate (Fru26P_2_). The cofactors NADH, NAD, ATP, ADP, phosphate, UTP and UDP are not treated as dynamic variables. All physiological metabolites produced or consumed in the hepatocyte during glycolysis and gluconeogenesis are comprised into lactate. Reproduced from [23], adapted from [67]. (B) sinusoidal unit describing blood flow, nutrient and hormone distribution within the sinusoids. The model encompasses the blood vessel, the adjacent space of Disse and the surrounding hepatocyte cell layer. It is described by morphological parameters (blood vessel radius, thickness of the space of Disse, hepatocyte thickness, hepatocyte number, sinusoid length, degree of fenestration) and systemic parameters (central and portal vein hydrostatic pressure, plasma and lympgh oncotic pressure, diffusion coefficients).

The reaction scheme for the glucose metabolism of a single hepatocyte is depicted in Fig 1A. It comprises the pathways for glycolysis, gluconeogenesis, glycogen synthesis and degradation and the three cellular compartments of cytosol, endoplasmic reticulum and mitochondrion (see Fig 1A). The time-dependent variations of metabolite concentrations are governed by ordinary differential equations. The rate laws for enzymes and membrane transporters were either taken from the literature or constructed on the basis of published experimental data. The rate equations for the metabolic enzymes explicitly take into account substrate regulation, allosteric regulation as well as activity changes due to reversible phosphorylation in response to insulin and glucagon signalling. Model input comprises extracellular nutrient and hormone concentrations as well as cellular protein abundance. The detailed rate laws describing the metabolic part of the model are given in S1 Supplement. The model is based on a previously published model of hepatic carbohydrate metabolism [23].

#### Parameterization of the metabolic model

The kinetic parameters of metabolic enzymes were taken from the literature. For each parameter, the experimental source is cited in supplements 1 and 2. If for an enzyme-kinetic parameter more than a single measurement was available, we used one representative value that fits with the majority of the reported values and that—whenever possible—was obtained in an enzyme assay that reported consistent values for other kinetic parameters. Mathematical terms in the rate law related to allosteric enzyme effectors which are not included in the model were neglected. Their average contribution is thus indirectly contained in the fitted Vmax values. The only free adjustable parameters of the model are the maximal enzyme activities (Vmax) which depend on the expression level of the enzyme protein and numerous other factors (cellular milieu, ligands not included into the model or even unknown). Vmax values were determined by adjusting simulated steady-state and time-dependent metabolite profiles and flux distributions to experimental data obtained with isolated hepatocytes in culture or the perfused liver (details are given in [23]).

#### Blood perfusion and tissue transport

The tissue transport of water, nutrients and blood is described by a compartment model. within the sinusoid by a compartment model. The STU is dissected into N discrete adjacent domains where N is the number of hepatocytes. Each domain subdivides into three compartments representing a single hepatocyte and the spatially associated part of the sinusoid and the space of Disse. Within the 3N compartments the concentrations of metabolites and hormones are constant. The exchange of metabolites and hormones between adjacent compartments are given by the following elementary processes: (i) Pressure-dependent directional convective flow of blood within the sinusoid from the periportal to pericentral pole, (ii) diffusion within the sinusoid, (iii) pressure-dependent directional convective flow from the sinusoid lumen to the space of Disse, (iv) diffusional exchange between the sinusoid lumen and space of Disse, (v) pressure-dependent directional convective transport of fluid in the space of Disse, (vi) diffusion within the space of Disse, (vii) active transport of metabolites between the space of Disse and the cytosol of hepatocytes.

Lateral blood flow in the vessel compartment is described by the Hagen-Poiseuille law for fluid flow through a cylinder. Water flow in the space of Disse is described by the Hagen-Poiseuille law for a hollow cylinder. Exchange of water between the vessel and the space of Disse is driven by hydrostatic and oncotic pressure difference between the blood vessel and the space of disse. The blood-born substances considered in our model are the metabolites glucose and lactate, the hormones insulin and glucagon and the indicator substances erythrocytes, albumin and water. The mathematical description of the tissue model and a complete list of all tissue parameters is given in S2 Supplement.

#### Hormonal regulation of metabolism

Hormonal regulation of metabolic fluxes is on the short term brought about by changes in the ratio of the phosphorylated and de-phosphorylated form of interconvertible metabolic enzymes. The phosphorylation states of the key regulatory enzymes of hepatic carbohydrate metabolism are controlled by the concentrations of the hormones insulin and glucagon. Insulin and glucagon concentrations in the plasma are determined by the glucose-dependent secretion rates of these hormones from the pancreas (ß- and α cells, respectively) and their plasma clearance. We used the same empirical functions developed in [23] to relate the plasma level of glucose to the plasma level of insulin and glucagon (= glucose-hormone transfer function, GHT) and to relate the plasma concentration of the hormones to the phosphorylation state of hormone sensitive enzymes (= hormone-phosphorylation transfer function, HPT).

Plasma concentrations of insulin and glucagon are usually determined in peripheral blood samples. However, pancreatic hormones released in the portal vein are cleared during their passage through the liver and the body which results in a gap between reported plasma concentrations and the true intra-hepatic hormone concentrations Comparison of measured hormone concentrations in the portal vein, central vein and systemic circulation revealed that the extra-hepatic body clearance accounts for about 50% of total hormone clearance [24]. We took this fact into account by setting the concentration values of insulin and glucagon in the incoming portal blood to the two-fold value of their plasma values.

Hepatic hormone clearance occurs preferentially through binding of the hormones to their respective receptors and subsequent internalization. We thus linearly scaled the rate of hormone clearance to the signaling strength of the hormone. A detailed description of the functions and their construction is given in S3 Supplement.

#### Main limitations and simplifications of the model

1. The main gluconeogenetic precursors lactate, external and internal amino acids and glycerol are lumped together into one generic precursor "lactate"
2. ATP producing and utilizing processes are not included, the adenine nucleotides ATP, ADP and AMP, the uridine nucleotides UTP and UDP and inorganic phosphate Pi are kept constant at their normal values. The same holds for the pyridine nucleotides NAD and NADH.
3. The contribution of monosaccharides other than glucose (fructose, galactose) to the hepatic carbohydrate metabolism is not considered.
4. Coupling of oxaloacetate (OA) with reactions of the citric acid cycle (formation by the malate dehydrogenase, utilization by the citrate synthase) is not included
5. Branching of the glycolytic triose phosphates (DHAP) to the lipid precursor glycerol phosphate is neglected
6. The representation of the sinusoid as a straight tube aligned by a single layer of linearly arranged cells is certainly a gross simplification of the true microanatomy of the liver.

#### Model simulations

With 20 hepatocytes per STU, the model is given by a system of 889 first-order differential equations which was numerically integrated by using the ODE15S solver of the MatLab package, Release R2011b, The MathWorks, Inc., Natick, Massachusetts, United States. The numerical simulation yielded stable solutions at variable number of cells (15–25) and variable parameter values.

For the interested reader, an SBML file of the cellular metabolic model is available from the authors.

### CT perfusion of the liver

#### CT data acquisition

The perfusion CT study was performed using a multi-slice scanner (SOMATOM Definition AS and Definition Flash Siemens Healthcare, Forchheim, Germany).The CT protocol consisted of a non-enhanced abdominal low-dose CT (40 mAs; 100 kV; SL, 5.0 mm; collimation, 128 0.6 mm; tube rotation time, 0.5 s; pitch, 0.6), which was obtained to localize the liver porta. Subsequently, a scan range of 6.9 cm z-axis coverage was planned over the involved liver, followed by a VPCT using an adaptive spiral scanning technique in shuttle mode. Perfusion parameters were: 80 kV; 100/ 120 mAs (for patients</>70 kg, respectively); collimation, 64 0.6 mm with z-flying focal spot; and 26 CT-whole coverages of the liver volume within a total scan time of 40 s. The mean radiation exposure for liver perfusion measurements was 7.5 mSv. Contrast medium was administered by using a dual-head pump injector (Stellant, Medtron, Saarbruecken, Germany). For this purpose, 50 mL Ultravist 370 (Bayer Vital, Leverkusen, Germany) were injected in all patients irrespective of patients’ weight at a flow rate of 5 mL/s in an antecubital vein followed by a saline flush of 50 mL NaCl at 5 mL/s. All images were transferred to an external workstation (Multi-Modality Workplace, Siemens, Erlangen, Germany) for analysis. An abdominal scan in portal vein phase was also obtained.

#### CT data analysis

All data sets were transferred to a dedicated workstation (Syngo MMWP, VE 36A, Siemens Healthcare, Forchheim, Germany) and quantitative data evaluation was performed with a commercial software (Syngo Volume Perfusion CT Body (Siemens Healthcare, Forchheim, Germany).

CT perfusion analysis software is based on the maximum slope model using time density curves (TDC) to determine perfusion. Calculation of arterial liver perfusion (ALP) and portal venous perfusion (PVP) according to the dual blood supply of the liver by hepatic artery and portal vein is done by using the time of peak splenic enhancement as a separation point of arterial and portal-venous phase by drawing region of interest (ROI) in portal vein and spleen respectively. Arterial TDC for ALP is calculated dividing maximum arterial slope by maximum aortic enhancement. Portal-venous TDC for PVP is calculated dividing maximum portal-venous slope by maximum portal-vein enhancement. Hepatic Perfusion index (HPI) in % represents ALP divided by the sum of ALP and PVP.

Automated motion correction and noise reduction of the datasets were applied by using an integrated motion correction algorithm with non-rigid deformable registration for anatomic alignment. Regions of interest (ROI) were placed in the abdominal aorta, portal vein, and spleen. For the HCC lesions quantitative perfusion parameters BF, BV, ALP, PVP and hepatic perfusion index (HPI) were obtained. Additionally, arterial liver perfusion (ALP), portal venous perfusion (PVP) and hepatic perfusion index (HPI) were registered also in the non-involved liver parenchyma at more sites including the right and left liver lobes. This was done drawing a Volume of interest (VOI); an automatic pixel based recognition algorithm allowed for encompassing whole tumor volume along the z-axis. Subsequently, a separate VOI was drawn encompassing the maximum perfused site within the tumor which was visually determined using the ALP-color coded map. BF, ALP and PVP is indicated as ml per 100ml of tissue per minute, BV is indicated as ml per 100ml of tissue and HPI is indicated in percent.

All data generated or analyzed during this study are included in this published article.

## Results

### Zonation of carbohydrate metabolism: Periportal and pericentral hepatocytes

**Fig 2:**
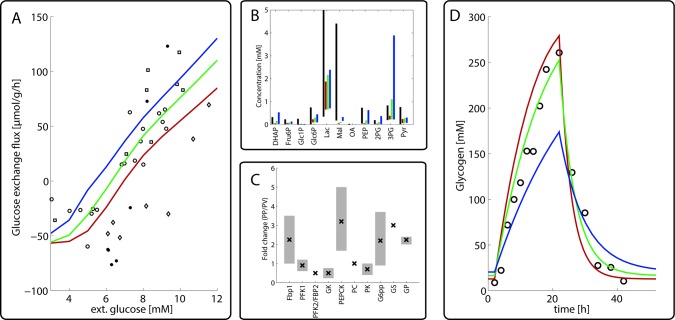
*Metabolic features of the periportal (PPH)*, *pericentral (PCH) and mean (MH) hepatocyte* (A) Simulated glucose exchange fluxes of the PPH (red), MH (green) and PCH (blue). Positive values of the glucose exchange flux correspond to net glucose uptake, negative values correspond to net glucose release. External glucose was varied between 3 and 12 mM. Experimental data were taken from [24, 26–28]. (B) Reported (black) concentration ranges of selected metabolites and simulated concentration values for the PPH (red), MH (green) and PCH (blue). Note that the experimental concentration values were obtained in liver homogenates or cultures of isolated hepatocytes and thus represent average values across different types of hepatocytes. Data were taken from various experimental sources [29–37]. (C) Average ratio of measured protein abundances in hepatocytes stemming preferentially from the periportal and pericentral region. Vertical lines indicate standard deviations. The circles indicate abundance ratios used to calibrate the model for the PPH and PCH. Experimental data are from various sources [6–21]. (D) Simulated glycogen concentration in PPH (red), MH (green) and PCH (blue) during a starvation-refeeding experiment. The initial state at t = 0 was obtained by simulating as 24h fasting period with a plasma glucose level of 4 mM. At t = 0 the plasma glucose level was elevated to 10 mM for 24 hours and then again reduced to 4 mM. Experimental data were taken from [25].

The various hepatocytes lining the sinusoids display a heterogeneous endowment with metabolic enzymes. Fig 2C shows the reported ratios of enzyme abundances in periportal and pericentral hepatocytes. For example, the abundance of the glycolytic enzyme pyruvate kinase (PK) was found up to 3-fold higher in hepatocytes isolated from the pericentral region compared to cells isolated from the periportal region. For the calibration of the model, it was necessary to define a 'mean hepatocyte' (MH) which best represents metabolic parameters determined for the whole organ. To this end we put the maximal enzyme activities (V_max_ values) of the MH to the arithmetic mean of the V_max_ values of the hepatocytes that are spatially closest to the portal and central pole (in the following referred to as periportal and pericentral hepatocytes, PPH and PCH). The ratios of the V_max_ values of the PPH and PCH were put to the measured ratios of protein abundances. All V_max_-values are given in S1 Supplement.

For the enzyme endowment of the individual hepatocytes lying between the PPH and PCH we assumed that the protein abundances of the key regulatory enzymes (see Fig 2C) change linearly from the portal to the central region, i.e. the protein abundance of the enzyme in the i-th hepatocyte is given by the relation

$$E\left(i\right)=E_{PP}+\frac{i-1}{N-1}\left(E_{PC}-E_{PP}\right)=E_{PP}\left(1+\frac{i-1}{N-1}\left[\alpha -1\right]\right)$$

where i numbers the spatial position of the hepatocyte along the sinusoid (i = 1 labels the hepatocyte closest to the portal pole, i = N labels the hepatocyte closest to the central pole), E_PP_ and E_PC_ are the enzyme abundances of the enzyme in the first and the last cell and α=EPCEPP is the ratio of enzyme abundances in periportal and pericentral cells. We assume that the metabolism of the whole liver can be best represented by the metabolism of a 'mean' hepatocyte at position i = N/2 which is endowed with the mean protein abundance (E_PP_+ E_PC_)/2. Hence, the Vmax values of the enzymes in this cell, V_max_ (N/2), were chosen such that that the best concordance between simulated metabolite concentrations and fluxes and measured values in the whole organ was achieved. Once a numerical value for V_max_ (N/2) is known, the V_max_ values of the enzyme in the other cells can be calculated from the relation

$$\frac{V_{\max}(i)}{V_{\max}(\frac{N}{2})}=\frac{E\left(i\right)}{E\left(\frac{N}{2}\right)}$$

because the maximal enzyme activity of an enzyme is up to a constant factor (the turnover rate constant) proportional to the protein abundance.

First we simulated the glucose exchange flux for the PPH, PCH and MH at plasma glucose concentrations in the physiological range between 3 mM and 12 mM (Fig 2A) and compared them to the experimentally determined whole liver glucose exchange fluxes (see [23]) for details). The feasibility of the calculated metabolic states was checked by the good concordance of calculated and experimentally determined ranges of metabolite concentrations (Fig 2B). The simulations show that the PPH has a higher capacity for gluconeogenesis, while the PCH has a higher capacity for glycolysis for each plasma glucose concentration. The set-point, i.e. the plasma glucose concentration at which net glucose exchange flux of the liver equals zero, is shifted from 7.0 mM for the PPH to 5.4 mM for the PCH. Notably, we found a similar set-point shift between hepatocytes in the fasted and fed state for the liver ([23]).

Next we simulated the dynamic changes of intrahepatic glycogen during a starvation-refeeding cycle (Fig 2D). Experimental data of the temporal glycogen content were taken from [25] where fasted rats were fed ad libitum for 20 hours and subsequently starved. We started the simulation with a pre-fasted liver (plasma glucose concentration of 4 mM). Interestingly, both the PPH and the PCH display smaller variations of the glycogen pool compared to the MH.

### Simulation of indicator dilution curves

**Fig 3:**
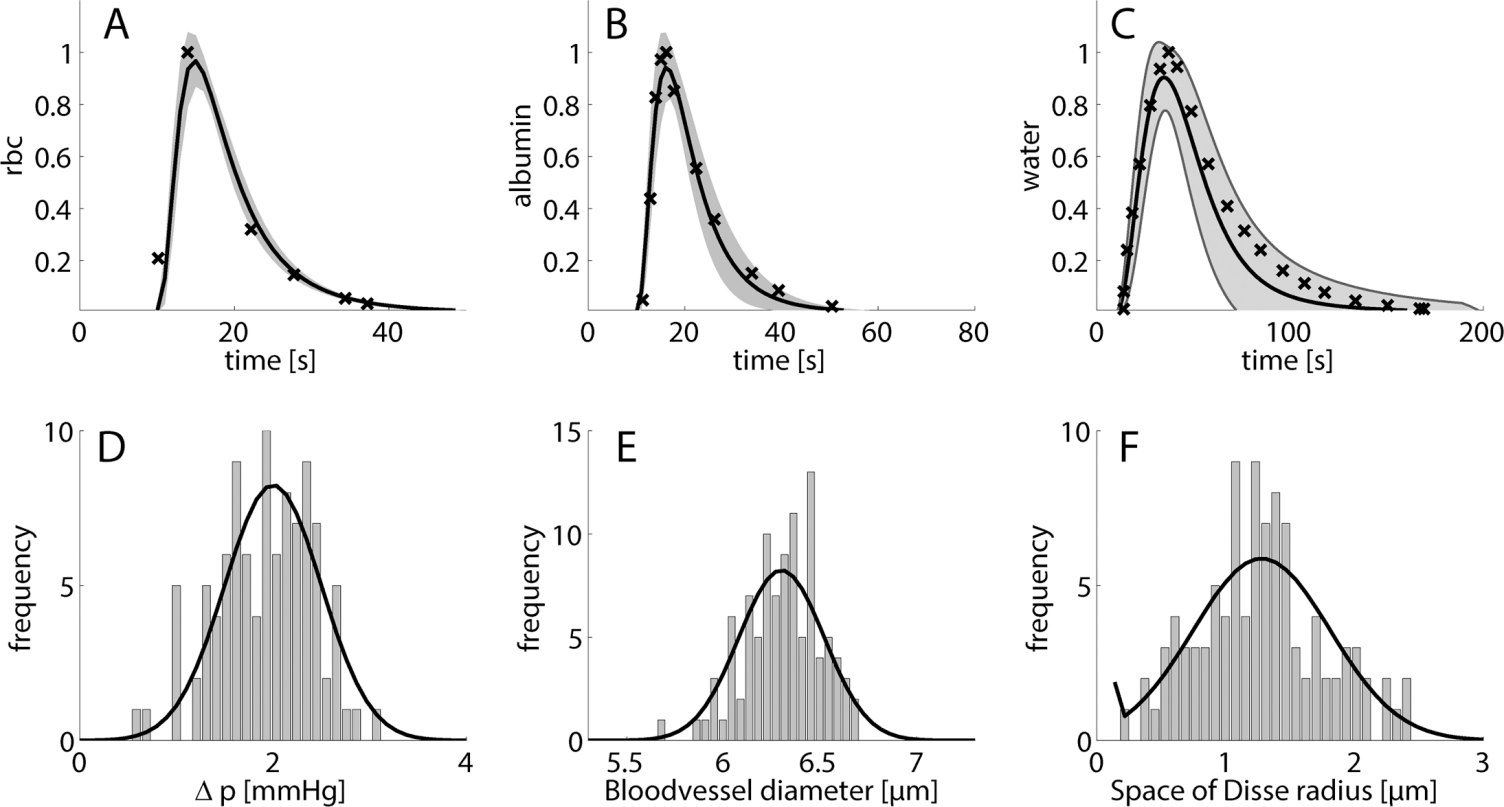
*Effect of variations of tissue parameter on indicator dilution curves* Simulations were run 100 times with random structural parameter sampling. Sampled parameters include portal-central pressure difference, blood vessel diameter, thickness of space of Disse, hepatocyte radius, cell number along the sinusoid, degree of fenestration. (A) indicator dilution curve for labeled red blood cells; (B) indicator dilution curve for labeled albumin; (C) indicator dilution curve for labeled water. Crosses depict experimental data taken from [38], black line represents the mean of 100 simulations, gray shaded areas represent standard deviations. Parameter distribution for portal-central pressure difference (D), blood vessel diameter (E) and thickness of space of Disse (F). Data taken from [39–41].

For the calibration of the blood flow model we compared calculated time courses of various indicators with the experimental data obtained by the indicator dilution technique in an in situ perfused phenobarbital-treated rat liver [34] (Fig 3A–3C). A soluble indicator substance is injected into the portal vein and the temporal concentration change in the central vein (= indicator dilution curve) is measured. Depending on blood flow velocity, diffusional exchange rates and the accessible distribution space, different indicators give rise to different dilution curves. Indicators used for model calibration were red blood cells (RBC), albumin and water. Since RBCs are larger than the size of the fenestrae, they are confined to the vascular compartment. Albumin is small enough to penetrate the fenestrae and to enter the space of Disse, but the endocytotic uptake rate of albumin into hepatocytes is negligible within the short time window (1–2 minutes). Water can enter the space of Disse and the cellular compartment.

From the average length of sinusoids (≈ 300 μm) and the mean blood flow velocity one can estimate that the transition time of red blood cells trough a liver sinusoid is about 1 second. However, the experimentally determined dilution curve for red blood cells has a width of about 40 seconds. This delay is due to the time required by the red cells to be transported from the injection site to the sinusoids and from the sinusoids to the collecting veins. To take this into account, we approximated the temporal plasma profile of all indicators by the mean dilution curve of the red cells.

To take into account random variations in tissue structure and blood pressure, we simulated indicator dilution curves for a multitude of liver sinusoids while randomly sampling the trans-sinusoidal pressure difference Δp and the structural parameters vessel diameter and thickness of the space of Disse from the respective observed distributions (see Fig 3D–3F). The largest variability of the indicator dilution curves was obtained for labeled water where the half-life decay time varied between 50–70 seconds. The smallest variability resulted for the dilution curves of red cells.

### Hepatic glucose exchange and hormone clearance at the tissue scale

During the passage of blood through the liver, the plasma concentrations of glucose and hormones are continuously changing. We simulated the intra-sinusoidal alterations in plasma glucose and hormone concentrations at different fixed portal glucose concentrations in the physiological range of 3–15 mM. The corresponding portal hormone concentrations were calculated by means of the glucose-hormone transfer (GHT) function (see S3 Supplement). The concentration of plasma lactate, which in our model represents all gluconeogenetic precursor substrates, was kept at 2 mM. Simulations were run until a steady state was reached.

The simulations were repeated 100 times with randomly and independently sampled structural, morphological and metabolic parameters. Structural and morphological parameters were drawn from their measured distributions (Fig 3D–3F), while protein abundances were sampled from equal distributions reflecting the variability of the periportal to pericentral protein ratios (Fig 2C).

**Fig 4:**
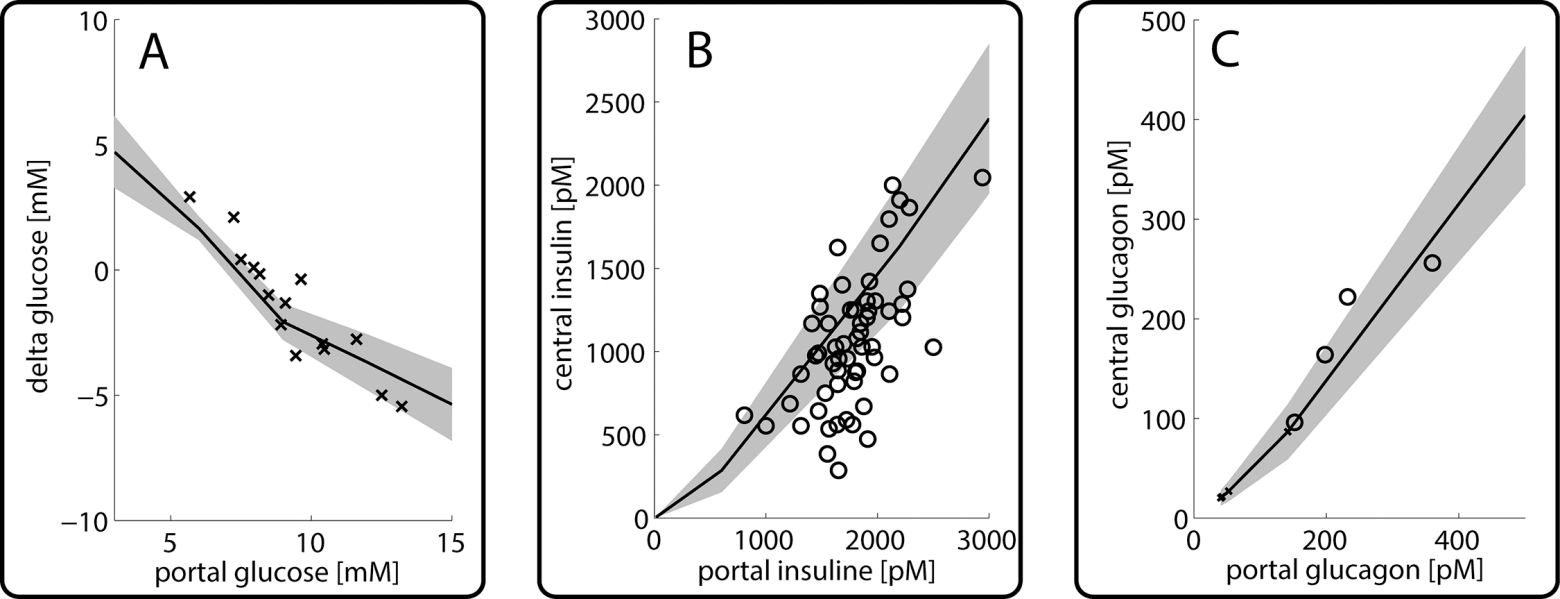
*Hepatic porto-venous glucose and hormone concentrations differences* (A) Porto-venous concentration difference of plasma glucose concentration as function of the portal glucose concentration. Experimental data from [42]. (B) Insulin concentration in the central vein in dependence of portal insulin concentration. Experimental data from [24]. (C) Glucagon concentration in the central vein in dependence of portal glucagon concentration. Experimental data from [43].

A convenient measure to assess the contribution of the liver to the homeostasis of the plasma glucose is the portovenous glucose concentration difference. The mean and variance of the computed and the clearance of the hormones insulin and glucagon are shown in Fig 4. In agreement with the experimental data, the model simulations predict that the liver acts as glucose producer for portal plasma glucose concentrations below 8 mM, whereas it turns to a glucose consumer for portal glucose concentrations higher than 8mM. The hormone extraction curves in Fig 4B and 4C show that during one passage a fraction of 20–40% glucagon and 30–80% of insulin can be removed by the liver. These values are also in good agreement with experimental findings [20].

Interestingly, the set-point for the STU lies at approximately 7.5 mM and is therefore even higher than the set-points of the isolated periportal and pericentral hepatocyte (see Fig 2A). While the set-points for the individual hepatocytes were calculated with the GHTF given in appendix 3, the relation between plasma glucose and plasma hormones changes along the portal-central axis as glucose is exchanged (either taken up or produced) and hormones are gradually cleared. Therefore the actual relation between glucose and hormones (and thereby the position of the set-point) changes from cell to cell and eventually determines the set point of the STU as a whole.

**Fig 5:**
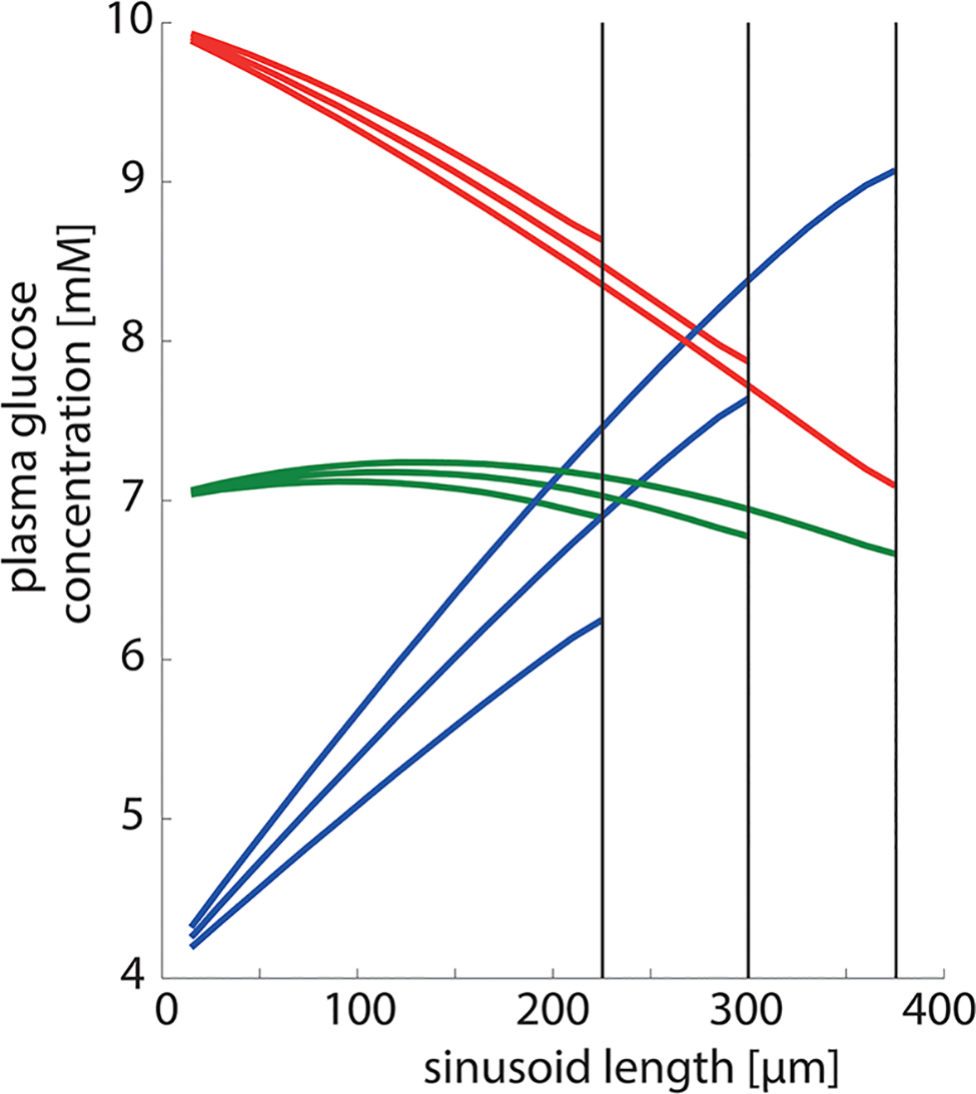
Effect of sinusoidal length on STU functionality. Sinusoidal length was 375 μm /300 μm/ 225 μm corresponding to 25/20/15 hepatocytes. Portal plasma glucose concentrations were 10 mM (red lines), 7 mM (green lines) and 4 mM (blue lines) corresponding to fed, normal and fasted state.

The grey-shaded areas in Fig 4 indicate the range of variability in the arteriovenous glucose difference for a single STU as caused by random variations of tissue parameters. In the fibrotic and cirrhotic liver, striking structural changes are an enlargement of hepatocytes ([44]) and a decrease in the effective length of sinusoids (mainly due to sinusoidal capillarization ([45]). We thus simulated how the metabolic input-output relation of a single STU is affected by the length of the sinusoid (see Fig 5). This analysis revealed that the length of the sinusoid has severe consequences for the functional output. The longer the sinusoidal length–and thereby the contact time for the exchange of glucose–the higher the alteration in plasma glucose concentration. At high glucose levels a longer sinusoid clears more glucose form the blood (red lines), while at low glucose levels a longer sinusoid produces more glucose (blue lines). If the plasma glucose is close to the set point, the sinusoidal length is not a major determinant for glucose exchange (green lines).

**Fig 6:**
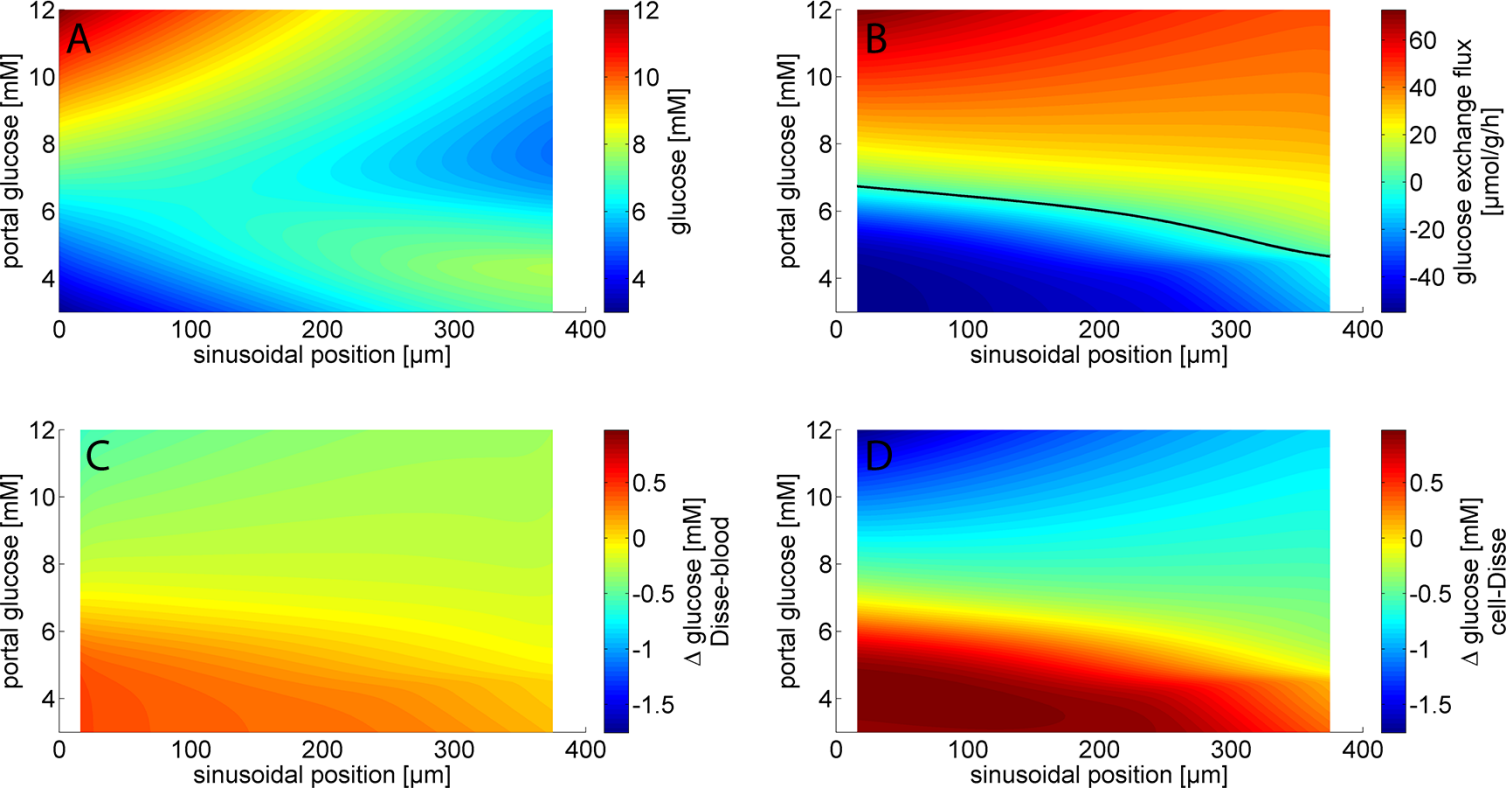
Glucose distribution at different spatial positions within the STU. Mean values from 100 simulations with randomly sampled STUs are shown. The STU is made up by 22 hepatocytes arranged along a sinusoid with a length of 200 μm. Portal glucose concentrations were varied between 3 and 12 mM. The continuous lines were created by linear interpolation between the discrete values (one value per compartment) obtained in the model simulation. (A) Glucose concentration; (B) Glucose exchange fluxes; (C) Glucose concentration differences between the space of Disse and the sinusoid glucose gradient; (D) Glucose concentration differences between the space of Disse and hepatocytes.

We further analyzed the distribution of glucose within the various compartments of the STU and the contribution of individual hepatocytes to the net glucose balance. Depending on the portal glucose concentration, the glucose concentration along the sinusoid decreases or increases in a nonlinear manner (Fig 6A). Intriguingly, for portal glucose concentrations in the range between 6 and 8 mM, upstream hepatocytes in the region around the portal field act as glucose producer (= positive glucose exchange flux) whereas downstream hepatocytes closer to the central pole act a glucose consumers (Fig 6B). This gives rise to an intra-sinusoidal glucose cycle where the glucose produced by the upstream hepatocytes are reutilized by the downstream hepatocytes.

As shown in Fig 6C, the glucose concentration gradient between the space of Disse and the sinusoidal lumen is largest at high portal glucose concentrations. But even in this situation the gradient remains below 1 mM. Thus, diffusion through endothelial fenestrae is sufficiently fast to prevent larger concentration gradients. Somewhat larger concentrations differences of up to 2 mM may occur between the space of Disse and the cytosol of hepatocytes (Fig 6D).

### Zonated glycogen variations during a starvation-refeeding cycle

**Fig 7:**
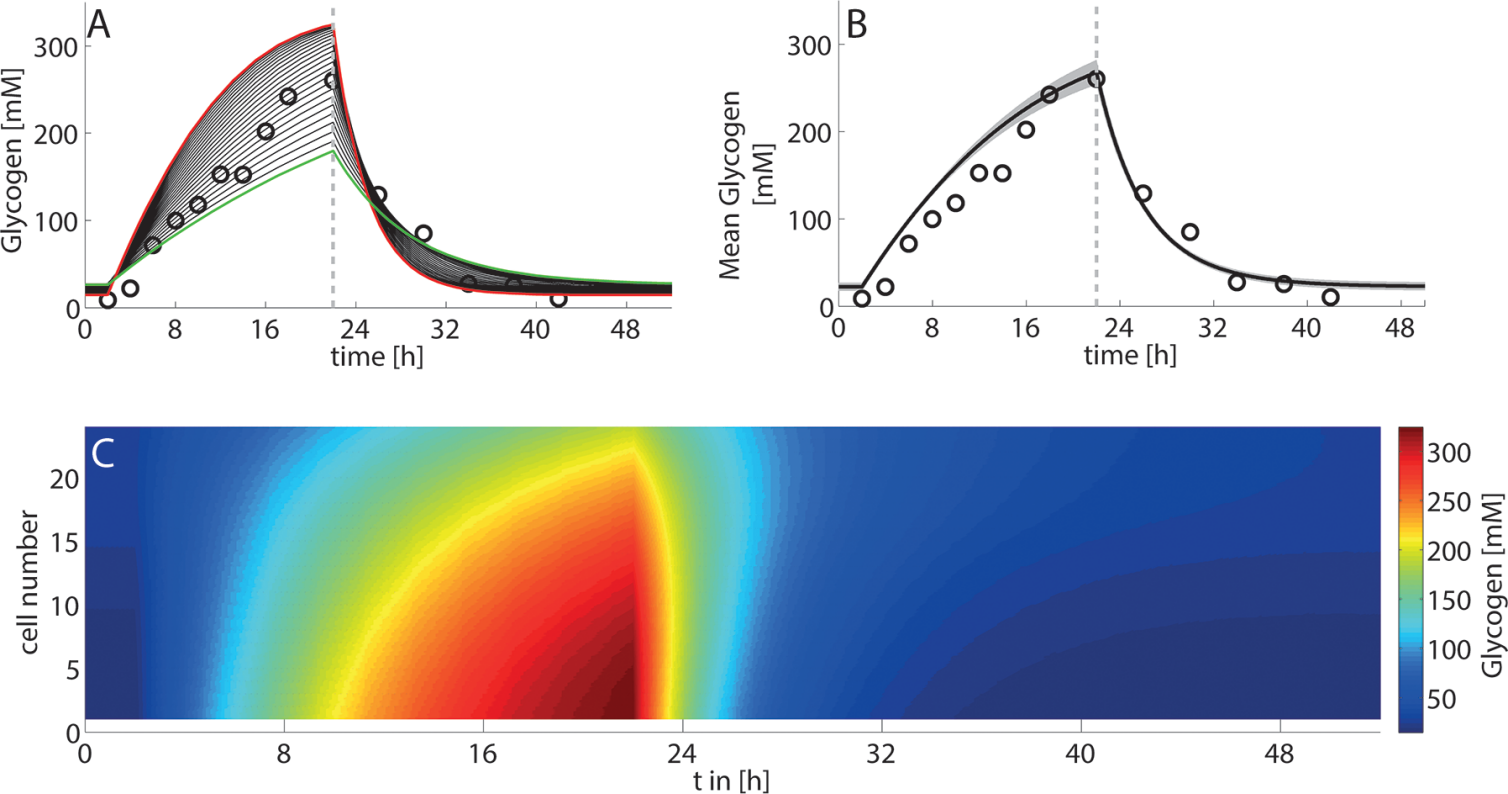
*Variations of the cellular glycogen content during a 48h fasting-refeeding cycle* (A) Simulated glycogen content of individual hepatocytes along the liver sinusoid. Red line–PPH (cell#1), green line–PCH (cell#22). Experimental data (open circles) were taken from [25]. (B) Simulated mean hepatic glycogen content (black line) and standard deviation (grey-shaded area) in response to parameter variation (100 trials with random parameter sampling based on the parameter distribution functions shown in Fig 3D–3F). (C) Simulated distribution of glycogen content across individual hepatocytes over time.

Next, we simulated the impact of a starvation-refeeding cycle on the dynamic changes of the glycogen content in the individual hepatocytes of the STU (Fig 7). As for the single cell case (Fig 2D), the simulation started with a pre-fasted liver (portal plasma glucose concentration of 4 mM) where the glycogen stores of all cell are almost completely emptied. During the feeding phase, the portal glucose concentration is increased to 8 mM, before it is reset to 4 mM during the fasting period. Simulations were repeated 100 times, again randomly and independently varying structural and morphological parameters as well as metabolic enzyme abundances from their respective distributions (see Fig 3, Fig 2 and S1 Supplement).

The amplitude of variations of the cellular glycogen content decrease from PPH to the PCH. In the PPH, the synthesis and degradation of glycogen PPH occurs with a significantly higher rate compared to the PCH. This implicates for hepatocytes of the periportal zone a higher glycogen content under euglycemic conditions but lower glycogen content in hypoglycaemia.

### Sensitivity analysis of the porto-venous glucose difference

**Fig 8:**
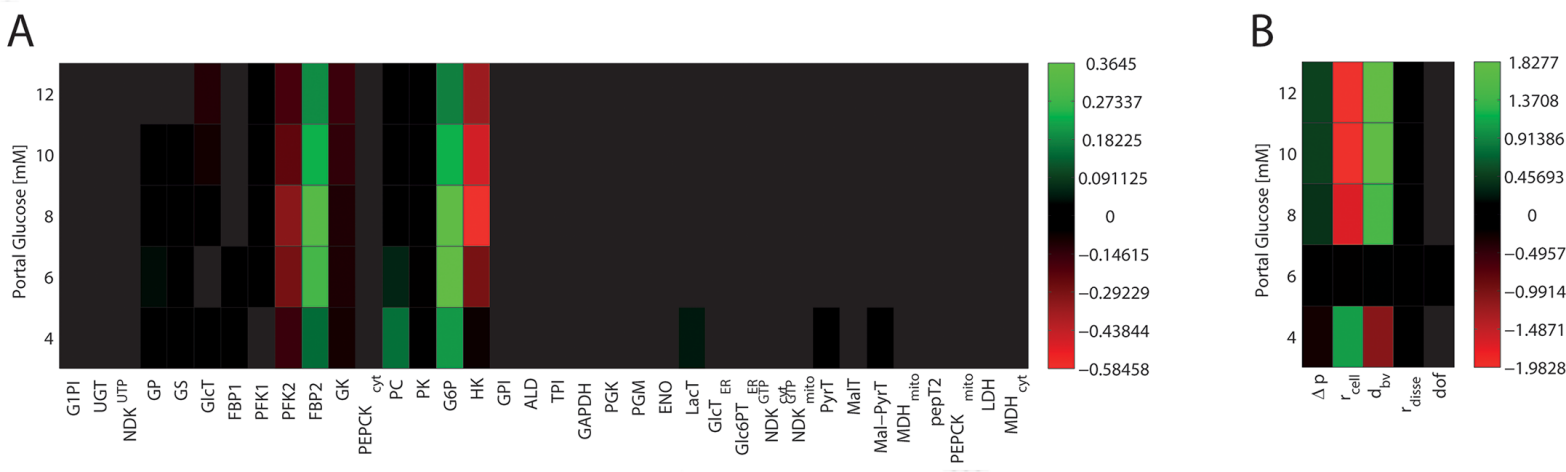
*Sensitivities* for metabolic enzymes (A) as well as structural and systemic parameters (B) with respect to central glucose concentration for different plasma glucose concentrations.

To check the relative influence of metabolic parameters and tissue parameters on the performance of the STU, we carried out a sensitivity analysis of the model (Fig 8). The sensitivity S(p) was quantified by the change of the hepatic arteriovenous glucose difference ΔGlu_PV_ elicited by a small (= 5%) change of the model parameter p (enzyme activity, tissue parameter, capillary blood pressure), i.e.

$$S(p)=\frac{p}{\delta p}\frac{\delta \;\Delta v_{PV}}{\Delta v_{PV}}=\frac{1}{\varepsilon }\left[\Delta v_{PV}\left(p+\varepsilon p\right)-\Delta v_{PV}\left(p-\varepsilon p\right)\right]$$

with *ε* = 0.05.

The relative impact of individual metabolic enzymes is dependent on the metabolic state (fasted, fed), similar as found in [23] for the single hepatocyte. In contrast, the influence of structural parameters is less dependent on the metabolic state. The size of hepatocytes and the diameter of the sinusoids turned out to have the largest impact on ΔGlu_PV_. Intriguingly, the sensitivity analysis shows that variations of the tissue architecture have an equally important influence of the metabolic performance of the liver than variations of protein abundances.

### Regional metabolic differences due to inhomogeneous blood perfusion of the liver

The blood flow within different regions of the liver may vary up to a factor of three (see e.g. [46, 47]). We used our model to study the metabolic consequences of such variations of the regional blood flow. Blood flow was measures by perfusion CT (see Methods).

**Fig 9:**
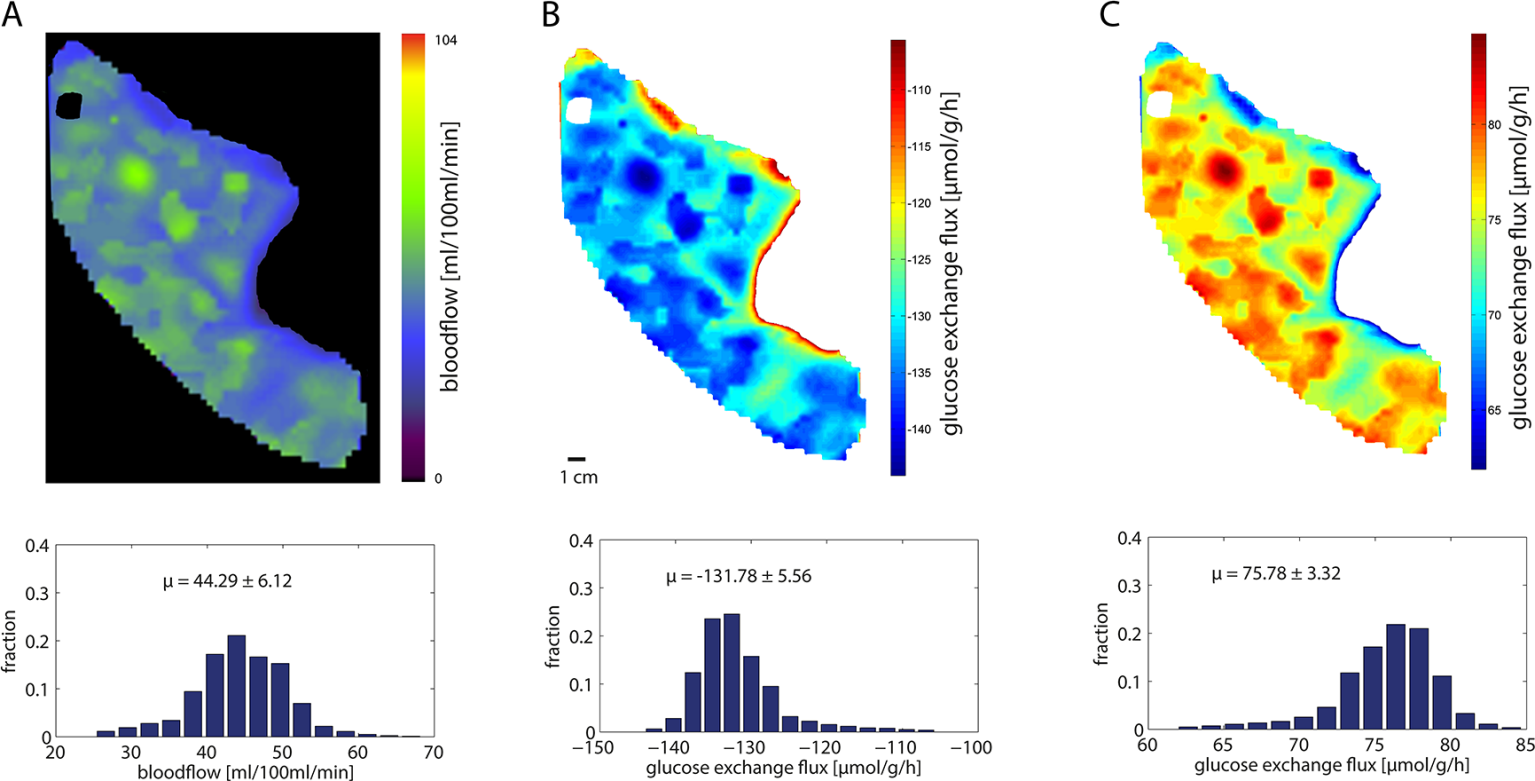
*Influence of the regional blood flow on the regional glucose uptake/production rate of the human liver* A Regional distribution of blood flow in a normal human liver assessed by perfusion CT. The histogram below shows the fractional distribution of blood flow values. The mean blood flow amounts to μ = 44.3 ml/100ml/min. *B* Simulated regional glucose exchange flux at a glucose plasma concentration of 4 mM (hypoglycemic condition) using the regional blood flow values shown in A as model input for the tissue model. The measured blood flow in a volume element of 100 ml was treated as the integral blood flow through 1.5 · 10^9^ sinusoids. This estimate was based on an average liver volume of 1.5 liter, an average number of 1.5 million lobules and an average number of 1000 sinusoids per lobule. The histogram below shows the fractional distribution of glucose exchange flux values. Note that negative flux values indicate glucose release. The mean glucose production rate of the liver amounts to μ = -131,8 μmol/g/h. C Simulated regional glucose exchange flux at a high glucose plasma concentration of 10 mM (hyperglycemic condition) using the regional blood flow values shown in A as model input for the tissue model. The mean glucose uptake rate of the liver amounts to μ = 75.8 μmol/g/h.

Fig 9 shows the CT—perfusion data and the corresponding model-based regional glucose production or utilization rate of a normal human liver. The blood flow through the sinusoid of the STU representing a small volume of liver was obtained by dividing the blood flow assessed by the perfusion CT through number of sinusoids 1.5 ∙ 10^9^ (sinusoids per 100 ml liver volume, see legend to Fig 9).

The average liver perfusion rate in this example was 44.3 ml/100ml/min with a standard variance of 6.1 ml/100ml/min. The computed regional glucose production rates (hypoglycemic case: portal glucose concentration set to 4 mM) and glucose uptake rates (hyperglycemic case: portal glucose concentration set to 10 mM) add up to a whole liver uptake rate of—131.8 μmol/g/h and production rate of 75.8 μmol/g/h, respectively. Interestingly, the variance of the glucose exchange fluxes is smaller than the variance of the blood flow values (compare the histograms in Fig 9A with those in Fig 9B and 9C). This is a consequence of the reciprocal relationship between blood flow rate and the exchangeable fraction of metabolites between blood and tissue. A decrease of the regional blood flow reduces the venous output volume but on the other hand increases in the leaving blood the concentrations of metabolites secreted by hepatocytes and increases the concentration of metabolites cleared by hepatocytes owing to the increased time span available for the exchange of blood metabolites with the tissue. Hence the mass output (= flow volume x concentration) is moderately buffered against changes of the blood perfusion rate.

## Discussion

Tissue level models are becoming an emerging tool for enabling clinical translational research [48]. In this work, we developed a multi-scale model of the sinusoidal tissue unit of the liver defined by a single sinusoid that is surrounded by the space of Disse and adjacent monolayers of hepatocytes. We used the model to investigate how the metabolic performance of the liver to ensure homeostasis of the systemic glucose metabolism is affected by zonated expression of metabolic enzymes, blood perfusion of the organ and metabolic coupling of hepatocytes via gradients of metabolites and hormones in the capillary blood stream.

Up to now only few models of liver metabolism exist which explicitly take into account the heterogenous intraacinar expression of metabolic enzymes and the metabolic implications of nutrient and hormone gradients along the portal-central axis. Ohno et al. [49] studied how the heterogenous expression of enzymes of the urea cycle and the glutamine synthetase may impact on the overall hepatic detoxification of ammonia. Their model subdivides the space between periportal blood entry and pericentral blood exit into 6 compartments. Compartment models with a similar architecture have been constructed for pharmacological applications, i.e. understanding the molecular basis for differential uptake and metabolization of xenobiotics in different zones [50, 51]. The most advanced zonated metabolic model of the liver has been published by Ashworth et al. [52]. They also used an 8- compartment model of the sinusoidal unit but on top included into their model 3 additional compartments representing the release of insulin from the pancreas and the exchange of glucose and fatty acids between the circulation and extra-hepatic tissues. The focus of this model was on the zonated lipid hyper-accumulation in case of non-alcoholic steatosis (NAFLD) caused by insulin resistance. A much simpler skeleton model addressing also the intriguing question of dispersed hepatic lipid accumulation has been recently proposed by Schleicher et al. [53]. Based on a 3-compartment model of the sinusoid they found that even in the absence of zonated enzyme expression the gradient of oxygen and fatty acids along the sinusoid may cause a zonated accumulation of lipid. In agreement with findings of the more advanced model of (Ashworth) their model suggests the zone-specific differences in the kinetic properties of the fatty acid uptake into hepatocytes are a key factor controlling the predominant accumulation of lipid in the pericentral region.

In the first part of this study, we analyzed how changes in the abundance of key metabolic enzymes reported for different zones of the liver acinus may affect the glucose exchange flux of the hepatocyte. As shown in Fig 2, zone-dependent expression of key regulatory enzymes of the glycolytic and gluconeogenetic pathway gives rise to significant differences in the relationship between plasma glucose level and the glucose exchange flux and glycogen turnover. Our calculations reveal a shift of the glucose-response curves for the periportal and pericentral hepatocyte in that periportal hepatocytes are stronger glucose producers whereas pericentral hepatocytes are more engaged in glucose utilization.

The signaling pathways responsible for the differential expression of metabolic enzymes in hepatocytes that are resident in different local positions of the sinusoid still remain unclear. Initially, the oxygen gradient was considered to be the most important driving force [54, 55]. Furthermore, the fact that incubation of isolated hepatocytes with high concentrations of either insulin or glucagon invokes a metabolic phenotype that resembles that of an periportal or pericentral hepatocyte [16] points to an important role of these hormones in the regulation of zonated gene expression. More recently, the Wnt/β-catenin developmental pathway has been shown to play a key role in the functional heterogeneity between periportal and pericentral mouse hepatocytes [56]. The Wnt pathway induces the genetic program of the pericentral hepatocyte and represses the genetic program of the periportal hepatocyte. On the contrary, the MAPK/ERK pathway seems to act in a reciprocal manner to counterbalance Wnt signaling and favors a periportal genetic program [57]. Also, a cross-talk between the transcription factor Hnf4a(alpha) and Wnt signaling has been proposed as a potential mechanism of liver zonation [58]. Comparing the protein ratios between periportal and pericentral hepatocytes to adaptive changes occurring in livers under extreme physiological settings like starvation, overnutrition or diabetes (see [23]) it becomes evident that the protein ratios between perioportal and pericentral hepatocytes are similar to the protein ratios observed between fed and starved or normal and diabetic liver. This indicates that the same mechnisms ensuring adaptation of the whole organ to variable nutrient and hormone challenges are responsible for liver zonation.

Heterogeneity occurs among liver lobules regarding their enzymatic endowment as well as in their geometric parameters (such as sinusoidal length and width, width of the space of Disse and degree of fenestration) and systemic parameters (such as central and portal vein hydrostatic pressures or plasma and lymph oncotic pressure). To assess the functional variability introduced by variances in these parameters, we conducted a multitude of simulations by randomly and independently varying structural, morphological and metabolic parameters. The simulations in Fig 4 show that on the microscopic scale, variability of metabolic and tissue parameters may cause variations of ΔGlu_PV_ of about 25%.

Interestingly the set-point for the STU lies at approximately 7.5 mM and is therefore higher than the set-point for the periportal and pericentral hepatocyte alone. While the set-points for the individual hepatocytes were calculated with the GHTF given in appendix 3, the relation between plasma glucose and plasma hormones changes along the sinusoid as glucose is exchanged (either taken up or produced) and hormones are degraded. Therefore the actual relation between glucose and hormones (and thereby the position of the set-point) for a cell within the STU (and the STU itself) does depend on the metabolic activity of the preceding cells resulting in the observed shift.

To better understand the influence of individual model parameters on the capability of the liver to act as an glucose homeostat, we calculated the sensitivity of ΔGlu_PV_ with respect to variations of each model parameter. This analysis revealed two important peculiarities in the control of the hepatic glucose metabolism. First, the impact of variances in the expression of metabolic enzymes on ΔGlu_PV_ is highly dependent on the metabolic state of the liver. For example, the enzyme pyruvate carboxylase exerts considerable control on ΔGlu_PV_ at low plasma glucose concentrations, but is unimportant at high glucose concentrations. This is in agreement with findings in [23], where we investigated the influence of metabolic enzymes under various pathological and physiological metabolic states. Second, the sensitivity of ΔGlu_PV_ towards variations of cell size, blood vessel diameter and pressure gradients are comparable to the largest sensitivity with respect to variations of enzyme activities, and this holds for every metabolic state studied (fasted, fed). Hence, alterations of tissue parameters as, for example, volume expansion of hepatocytes in the fatty liver, increased collagen density in the space of Disse in the fibrotic liver or arterioportal shunting of sinusoids in the cirrhotic liver, can be expected to have profound effects on the metabolic performance of the affected liver region. How such effects are possibly compensated by other healthy regions of the liver remains to be elucidated.

The model allows interesting insights into the intra-sinusoidal glucose distribution which is not directly accessible to experimentation. The average STU is able to transfer incoming plasma glucose concentrations over a wide physiological range of 3–15 mM to a narrow interval of 6–10 mM pericentral glucose concentration. This reflects the enormous capacity of the liver to regulate plasma glucose concentration over a wide range of physiological situations. The glucose output of the liver must not be confused with measured plasma glucose concentrations which normally lie around 5 mM to 6 mM. The difference arises from the glucose clearance by extrahepatic tissues, mainly muscle and brain. Importantly, the glucose gradient between the cells and the space of Disse is twice as large as between the space of Disse and the blood. Hence the exchange of glucose between the sinusoid and the space of Disse through endothelial fenestrae does not appear to be a rate limiting step. This is also reflected by the almost vanishing sensitivities against changes of the diameter of the fenestrae.

Relating our model simulations of the glycogen metabolism to experimental data it has to be noted that strikingly different glycogen distributions in the liver have been reported in the literature depending on the species (mice, rat, dog, human), assay (isolated hepatocytes, perfused liver, in vivo measurements), method of glycogen quantification (PAS staining, isotopomer analysis) and day-time of sampling. For example, Bartels et al. ([59]) found glycogen repletion in the perfused liver of fasted rats to be exclusively localized to PCH whereas an isotopomer analysis based on administration of [U-^13^C] glucose provided no evidence for zone-dependent differences in the glycogen repletion of 24h fasted conscious rats [60]. This makes it difficult to compare or simulations of the intra-acinar glycogen dynamics with experimental data. Both findings are in contradiction to our simulation which suggests a larger span and more rapid changes of glycogen in PPH compared with PCH (see Fig 2). However, this prediction is in perfect agreement with observations in [61] according to which glycogen withdrawal begins in zone 1, then spreads gradually to zone 3 and disappears after 12 hours if mice are fasted from 10.00 a.m. onwards. Intriguingly, the depletion pattern changes if fasting starts at midnight or at 10 p.m. In this case the glycogen is withdrawn evenly throughout the lobule. The authors speculated that circadian rhythms in the glycogen metabolism may account for these day-time dependent differences in the pattern of starvation-induced glycogen dynamics. Indeed, in Clock mutant mice the circadian oscillation of both the hepatic glycogen content and the circadian mRNA and protein expression of glycogen synthase 2 was severely dampened [62]. Moreover, the existence of a circadian rhythm of pancreatic insulin secretion in humans has been established [63]. We like to mention that shifting the HGR curve to higher or lower insulin secretion also shifts the glycogen distribution within the STU. Freinkel et al. ([64]) reported significantly higher insulin-to-glucose ratios in the morning than in the afternoon. Lambert and Hoet ([65]), studying seven obese and three non-obese females, found serum insulin levels to be higher during the night, when the subjects were not eating.

Our simulations shed some light on a novel mechanism which could be involved in the rapid adjustment of hepatic glucose metabolism to sudden changes of plasma glucose. As can be seen in Fig 6B, at portal glucose concentrations between 6 mM to 8 mM, glucose is produced in periportal hepatocytes and at the same time utilized in the pericentral hepatocytes. Such an intra-organ subdivision into glucose-producing and glucose-utilizing tissue regions is present in the kidney where the epithelial cells of the proximal tubule produce glucose along the gluconeogenetic pathways whereby glutamine serves as the major carbon source. The glucose-enriched blood reaches the cells of the distal tubule which utilize glucose in the glycolytic pathway for the production of ATP because oxidative phosphorylation is strongly limited by the low oxygen pressure in the distal region of the nephron. A similar function of the hepatic self-supply with respect to glucose seems not very likely because the oxygen gradient in the sinusoidal blood stream is only moderately changing from about 60 mmHg at the portal site to about 40 mmHg at the central site of the mice liver [66]. In the liver, the glucose cycle might establish a regulatory mechanism that allows a rapid increase of the glucose output by abruptly reducing the intra-hepatic glucose consumption. Hyperglycemia does not constitute an immediate threat for the organism as the adverse effects like protein glycosylation, diabetes, cardiovascular diseases, nephropathies or impaired vision develop over time periods of years. Contrary, short term hypoglycemia can have severe consequences. As the brain functionality relies almost exclusively on glucose as energy delivering substrate, even short hypoglycemic episodes of some minutes are accompanied by impaired vision, dizziness, unconsciousness or even death. To prevent the occurrence of hypoglycemic crisis, the liver produces in the periportal region more glucose than normally required for the plasma glucose homeostasis and extracts the surplus glucose in the pericentral region. By just reducing the intra-hepatic glucose consumption the glucose output could be rapidly elevated.

Finally, as first step towards a model-based construction of metabolic liver 'functiogram', we have used the model to convert the regional blood flow of the liver into regional glucose production- and utilization rates in hypo- and hyperglycemia, respectively. Interestingly, the metabolic heterogeneity appears to be smaller than the perfusion heterogeneity although there is still 25% - 42% variability in regional glucose exchange rates. The next step will be to apply our approach to unravel the regional metabolic capacity of steatotic, inflammatory, fibrotic and cirrhotic livers. This will require the re-parametrization of the tissue model with histological parameters of the diseased tissue and proteomics-based protein levels of metabolic enzymes. In combination with transient elastography and perfusion CT, such an *in silico* approach will provide a 3D *metabolic functiogram* of the liver of individual patients that provides valuable information on the available metabolic reserve capacity in distinct liver regions.

### Conclusions

In summary, we presented a model of sinusoidal glucose metabolism by extending a previously established model of hepatic glucose metabolism to include liver zonation. We developed a realistic sinusoidal blood flow model taking morphological and systemic parameters into account thereby bridging the scale from the cellular to the tissue level. Taking into account variations in the tissue parameters as well as variations in the enzyme abundance we assessed the variability of sinusoidal glucose input-output relationship occurring among different livers sinusoids thereby assessing inter-organ variability. Swelling, cirrhosis and restriction of blood flow contribute to progression of liver disease thus allowing for interesting future uses of the model as a valuable tool to analyze liver functionality especially under disease conditions.

## Supporting information

S1 Supplement Mathematical description of the metabolic model. (DOCX)

S2 Supplement Mathematical description of the blood flow model. (DOCX)

S3 Supplement Mathematical description of the hormone signaling model. (DOCX)
