# Supplementary material for: A multiscale modelling approach to assess the impact of metabolic zonation and microperfusion on the hepatic carbohydrate metabolism
Source: PLoS Comput Biol. 2018 Feb 15;14(2):e1006005. doi: 10.1371/journal.pcbi.1006005 (PMC5841820; doi:10.1371/journal.pcbi.1006005)
Supplement: S1 Supplement — (DOCX) [file pcbi.1006005.s001.docx]

# Stoichiometric matrix

$$\frac{d}{dt}DHAP= v_{ALD}-v_{TPI}$$

$$\frac{d}{dt}Fru16P_{2}= v_{PFK1}-v_{FBP1}-v_{ALD}$$

$$\frac{d}{dt}Fru26P_{2}= v_{PFK2}-v_{FBP2}$$

$$\frac{d}{dt}Fru6P= v_{GPI}-v_{PFK1}-v_{PFK2}+v_{FBP1}+v_{FBP2}$$

$$\frac{d}{dt}GAP= v_{ALD}+v_{TPI}-v_{GAPDH}$$

$$\frac{d}{dt}GDP= {-v}_{NDK^{GTP}}+v_{PEPCK}$$

$$\frac{d}{dt}GDP_{mito}= -v_{NDK^{GTP_{mito}}}+v_{PEPCK_{mito}}$$

$$\frac{d}{dt}Glc= v_{GLUT2}-v_{GK}+v_{GlcT_{ER}}$$

$$\frac{d}{dt}Glc_{dis}= {-v}_{GLUT2}\cdot\frac{Vol_{cell}}{Vol_{disse}}$$

$$\frac{d}{dt}Glc_{ER}= v_{G6P_{ER}}-v_{GlcT_{ER}}\cdot\frac{Vol_{cell}}{Vol_{ER}}$$

$$\frac{d}{dt}Glc1P= v_{GP}-v_{G1PI}-v_{UGT}$$

$$\frac{d}{dt}Glc6P=v_{GK}+v_{G6PT_{ER}}-v_{GPI}+v_{G1PI}$$

$$\frac{d}{dt}Glc6P_{ER}= -v_{G6P_{ER}}-v_{Glc6PT_{ER}}\cdot\frac{Vol_{cell}}{Vol_{ER}}$$

$$\frac{d}{dt}Glyc= v_{GS}-v_{GP}$$

$$\frac{d}{dt}GTP= v_{NDK^{GTP}}-v_{PEPCK}$$

$$\frac{d}{dt}GTP_{mito}= v_{NDK^{GTP_{mito}}}-v_{PEPCK_{mito}}$$

$$\frac{d}{dt}Lac= v_{LacT}+v_{LDH}$$

$$\frac{d}{dt}Lac_{disse}= -v_{LacT}\cdot\frac{Vol_{cell}}{Vol_{disse}}$$

$$\frac{d}{dt}Mal= v_{MalT}-v_{MDH}+v_{PyrMalT}$$

$$\frac{d}{dt}Mal_{mito}= -v_{MalT}\cdot\frac{Vol_{cell}}{Vol_{mito}}-v_{MDH_{mito}}-v_{PyrMalT}\cdot\frac{Vol_{cell}}{Vol_{mito}}$$

$$\frac{d}{dt}OA= v_{MDH}-v_{PEPCK}$$

$$\frac{d}{dt}OA_{mito}= v_{PC}-v_{PEPCK_{mito}}+{v_{MDH}}_{mito}$$

$$\frac{d}{dt}PEP= v_{EN}-v_{PK}+v_{PEPCK}-v_{PEPT}$$

$$\frac{d}{dt}PEP_{mito}= v_{PEPCK_{mito}}+v_{PEPT}\cdot\frac{Vol_{cell}}{Vol_{mito}}$$

$$\frac{d}{dt}13P2G= v_{GAPDH}-v_{PGK}$$

$$\frac{d}{dt}2PG= v_{PGM}-v_{EN}$$

$$\frac{d}{dt}3PG= v_{PGK}-v_{PGM}$$

$$\frac{d}{dt}Pyr= v_{PK}-v_{LDH}-v_{PyrT}-v_{PyrMalT}$$

$$\frac{d}{dt}Pyr_{mito}= v_{PyrT}\cdot\frac{Vol_{cell}}{Vol_{mito}}-v_{PC}+v_{PyrMalT}\cdot\frac{Vol_{cell}}{Vol_{mito}}$$

$$\frac{d}{dt}UDP= {-v}_{NDK^{UTP}}+v_{GS}$$

$$\frac{d}{dt}UDP\text{-}Glc= v_{UGT}-v_{GS}$$

$$\frac{d}{dt}UTP= v_{NDK^{UTP}}-v_{UGT}$$

# Reaction kinetics

## ALD (Aldolase)

$$Fru16P_{2}\leftrightarrow GAP+DHAP$$

$$v_{ALD}=v_{max}^{ALD}\cdot\frac{Fru16P_{2}-GAP\cdot{DHAP}/{k_{eq}^{ALD}}}{\left( 1+\frac{Fru16P_{2}}{k_{m}^{Fru16P_{2}}} \right)+\left( 1+\frac{GAP}{k_{m}^{GAP}} \right)\left( 1+\frac{DHAP}{k_{m}^{DHAP}} \right)-1}$$

$k_{eq}^{ALD}=$0.099 mM [[37](#_ENREF_37)]

$k_{m}^{Fru16P_{2}}=0.004$ mM [[38](#_ENREF_38)]

$k_{m}^{GAP}=$0.48 mM [[39](#_ENREF_39)]

$K_{m}^{DHAP}=0.38$mM [[39](#_ENREF_39)]

## EN (Enolase)

$$2PG\leftrightarrow PEP$$

$$v_{EN}=v_{max}^{EN}\cdot\frac{2PG-{PEP}/{k_{eq}^{EN}}}{1+\frac{2PG}{k_{m}^{2PG}}+\frac{PEP}{k_{m}^{PEP}}}$$

$k_{eq}^{EN}=$1.7 [[40](#_ENREF_40)]

$k_{m}^{2PG}=0.14 mM$ [[41](#_ENREF_41)]

$k_{m}^{PEP}=0.31 mM$ [[41](#_ENREF_41)]

## FBP1 (Fructose-1,6-bisphosphatase)

$$Fru16P_{2}\to Fru6P+P$$

$$v_{FBP1}=V_{max}^{FBP1}*\left( \left( 1-\gamma^{FBP1} \right)*v_{FBP1}^{native}+\gamma^{FBP1}*v_{FBP1}^{phospho} \right)$$

$$v_{FBP1}^{native}=\frac{Fru16P_{2}}{{Fru16P_{2}+k}_{m^{native}}^{Fru16P_{2}}}/ \left( 1+\frac{Fru26P_{2}^{n}}{{{(k}_{i}^{Fru26P_{2}})}^{n}} \right)/\left( 1+\left( \frac{AMP}{K_{i}^{AMP}} \right)^{n_{AMP}} \right)$$

$k_{m^{native}}^{Fru16P_{2}}=0.0029$mM [[42](#_ENREF_42)]

$k_{i}^{Fru26P_{2}}=0.00113$ mM [[42](#_ENREF_42)]

$n=1.26$ [[42](#_ENREF_42)]

$n_{AMP}=$2.43 [[42](#_ENREF_42)]

$K_{i}^{AMP}=$0.023 mM [[42](#_ENREF_42)]

$$v_{FBP1}^{phospho}=\frac{Fru16P_{2}}{{Fru16P_{2}+k}_{m^{phospho}}^{Fru16P_{2}}}/\left( 1+\frac{Fru26P_{2}^{n}}{{{(k}_{i}^{Fru26P_{2}})}^{n}} \right)/\left( 1+\left( \frac{AMP}{K_{i}^{AMP}} \right)^{n_{AMP}} \right)$$

$k_{m^{phospho}}^{Fru16P_{2}}=0.0019$ mM [[42](#_ENREF_42)]

$k_{i}^{Fru26P_{2}}=0.00113$ mM [[42](#_ENREF_42)]

$n=1.26$ [[42](#_ENREF_42)]

$K_{i}^{AMP}=$0.023 mM [[42](#_ENREF_42)]

$n_{AMP}=$2.43 [[42](#_ENREF_42)]

## GAPDH (Glyceraldehyde 3-phosphate dehydrogenase)

$$GAP+P+NAD^{+}\leftrightarrow13P2G+NADH+H^{+}$$

$$v_{GAPDH}=v_{max}^{GAPDH}*\frac{NAD^{+}\cdot GAP\cdot P-{13P2G\cdot NADH}/{k_{eq}^{GAPDH}}}{\left( 1+\frac{NAD^{+}}{k_{m}^{NAD^{+}}} \right)\cdot\left( 1+\frac{GAP}{k_{m}^{GAP}} \right)\cdot\left( 1+\frac{P}{k_{m}^{P}} \right)+\left( 1+\frac{NADH}{k_{m}^{NADH}} \right)\cdot\left( 1+\frac{13P2G}{k_{m}^{13P2G}} \right)-1}$$

$k_{eq}^{GAPDH}={10}^{-4}mM^{-1}$ [[43](#_ENREF_43)]

$k_{m}^{NAD^{+}}=$0.010 mM [[44](#_ENREF_44)]

$k_{m}^{GAP}=$ 0.035 mM [[44](#_ENREF_44)]

$k_{m}^{P}=$3.8 mM [[45](#_ENREF_45)]

$k_{m}^{NADH}=$ 0.006 mM [[45](#_ENREF_45)]

$k_{m}^{13P2G}=$ 0.01 mM [[44](#_ENREF_44)]

## GK (Glucokinase)

$$Glc+ATP\to Glc6P+ADP$$

$$v_{GK}=V_{max}^{GK}\cdot\frac{ATP}{{ATP+k}_{m}^{ATP}}\cdot\frac{({Glc)}^{n}}{\left( Glc \right)^{n}+{{(k}_{m}^{Glc})}^{n}}$$

$$V_{max}^{GK}=V_{0}^{GK}\cdot\frac{\left( Glc \right)^{n2}}{({Glc)}^{n2}+{{(k}_{a}^{Glc})}^{n2}}\cdot\left( 1-f\cdot\frac{Fru6P}{Fru6P+k_{i}^{Fru6P}} \right)$$

$n=$1.5 [[46](#_ENREF_46)]

$K_{m}^{Glc}=9 mM$ [[46](#_ENREF_46)]

$k_{m}^{ATP}=$0.55 $mM$ [[47](#_ENREF_47)]

$k_{i}^{Fru6P}=0.005 mM$ [[48](#_ENREF_48)]

$f= 0.75$ [[48](#_ENREF_48)]

$n2=3.7$ [[49](#_ENREF_49)]

$k_{a}^{Glc}=15.9 mM$ [[49](#_ENREF_49)]

## GlcT_ER_ (Glucose transport to ER)

$${Glc}_{ER}\leftrightarrow Glc$$

$$v_{{GlcT}_{ER}}=V_{max}^{{GlcT}_{ER}}*\frac{\left( Glc-{Glc}_{ER} \right)}{1+\frac{Glc}{k_{m}^{Glc}}+\frac{Glc_{ER}}{k_{m}^{Glc_{ER}}}}$$

$k_{m}^{{Glc}_{ER}}=1.37 mM$ [[50](#_ENREF_50), [51](#_ENREF_51)]

$k_{m}^{Glc}=1.22 mM$ [[50](#_ENREF_50), [51](#_ENREF_51)]

## GLUT2 (Glucose transporter 2)

$${Glc}_{disse}\leftrightarrow Glc$$

$$v_{GLUT2}=V_{max}^{GLUT2}\cdot\frac{{Glc}_{ext}-Glc}{1+\frac{{Glc}_{ext}}{k_{m}^{{Glc}_{ext}}}+\frac{Glc}{k_{m}^{Glc}}}$$

$k_{m}^{Glc}=17.3 mM$[[52](#_ENREF_52)]

$k_{m}^{{Glc}_{ext}}=17.3 mM$ [[52](#_ENREF_52)]

## **GP (Glycogen phosphorylase)**

$$Glyc+P\leftrightarrow Glc1P$$

$$v_{GP}=V_{max}^{GP}\cdot\left( \left( 1-\gamma^{GP} \right)\cdot v_{GP}^{native}+\gamma^{GP}\cdot v_{GP}^{phospho} \right)$$

$$V_{max}^{GP}=V_{0}^{GP}\cdot\left( \cdot\frac{Glyc}{store} \right)$$

$$store=375 mM$$

$$v_{GP}^{native}=V_{m{ax}_{native}}^{GP}\cdot\frac{Glyc\cdot P-{Glc1P}/{k_{eq}^{GP}}}{\left( 1+\frac{Glyc}{k_{m_{native}}^{Glyc}} \right)\cdot\left( 1+\frac{P}{k_{m_{native}}^{P}} \right)+\left( 1+\frac{Glc1P}{k_{m_{native}}^{Glc1P}} \right)-1}$$

$$V_{m{ax}_{native}}^{GP}=V_{0_{native}}\cdot\left( \frac{AMP}{AMP+K_{a_{native}}^{AMP}} \right)$$

$$V_{0_{native}}=\frac{1}{k_{m_{native}}^{Glyc}\cdot k_{m_{native}}^{P}}$$

$K_{a_{native}}^{AMP}=$0.36 mM [[53](#_ENREF_53)]

$k_{eq}^{GP}=0.21\left( mM \right)^{-1}$ [[55](#_ENREF_55)]

$k_{m_{native}}^{Glyc}=$ 2.5 mM [[54](#_ENREF_54)]

$k_{m_{native}}^{P}=500 mM$ [[54](#_ENREF_54)]

$$K_{m_{native}}^{Glc1P}=K_{0}^{Glc1P}\cdot\left( 1-\frac{AMP}{AMP+K_{a_{Glc1P}}^{AMP}} \right)$$

$K_{0}^{Glc1P}=250$ mM [[54](#_ENREF_54)]

$K_{a_{Glc1P}}^{AMP}= 0.5 mM$ [[54](#_ENREF_54)]

$$v_{GP}^{phospho}=V_{max_{phospho}}^{GP}\cdot\frac{Glyc\cdot P-{Glc1P}/{k_{eq}^{GP}}}{\left( 1+\frac{Glyc}{k_{m_{phospho}}^{Glyc}} \right)\cdot\left( 1+\frac{P}{k_{m_{phospho}}^{P}} \right)+\left( 1+\frac{Glc1P}{k_{m_{phospho}}^{Glc1P}} \right)-1}$$

$$V_{{max}^{phospho}}^{GP}=V_{0_{phospho}}\cdot\left( \frac{AMP}{AMP+K_{a_{phospho}}^{AMP}} \right)$$

$$V_{0_{phospho}}=\frac{1}{k_{m_{phospho}}^{Glyc}\cdot k_{m_{phospho}}^{P}}$$

$K_{a_{phospho}}^{AMP}=0.017$ mM [[53](#_ENREF_53)]

$k_{m_{phospho}}^{Glyc}=1.8 mM$ [[54](#_ENREF_54)]

$k_{m_{phospho}}^{P}=$ 2.1 mM [[54](#_ENREF_54)]

$k_{m_{phospho}}^{Glc1P}=0.7 mM$ [[54](#_ENREF_54)]

## **G**6PER (Glucose-6-phosphate phosphatase in the ER)

$${Glc6P}_{ER}\to{Glc}_{ER}+P$$

$${v_{G6P}}_{ER}=V_{max}^{{G6P}_{ER}}\cdot\frac{{Glc6P}_{ER}}{{{Glc6P}_{ER}+k}_{m}^{{Glc6P}_{ER}}}$$

$k_{m}^{{Glc6P}_{ER}}=$1.84 mM [[56](#_ENREF_56)]

$$V_{max}^{{G6P}_{ER}}=4.57\cdot{10}^{2} mM\cdot h^{-1}$$

## GPI (Glucose-6-phosphate isomerase)

$$Glc6P\leftrightarrow Fru6P$$

$$v_{GPI}=V_{max}^{GPI}\cdot\frac{Glc6P-{Fru6P}/{k_{eq}^{GPI}}}{1+\frac{Glc6P}{k_{m}^{Glc6P}}+\frac{Fru6P}{k_{m}^{Fru6P}}}$$

$k_{eq}^{GPI}=$ 0.3 [[58](#_ENREF_58)]

$k_{m}^{Glc6P}=0.055 mM$ [[59](#_ENREF_59)]

$K_{m}^{Fru6P_{cyt}}=$ 0.12 mM [[59](#_ENREF_59)]

## G1PI (Glucose-1-phosphate isomerase)

$$Glc1P\leftrightarrow Glc6P$$

$$v_{G1PI}=v_{max}^{G1PI}\cdot\frac{Glc1P-{Glc6P}/{k_{eq}^{G1PI}}}{1+\frac{Glc1P}{k_{m}^{Glc1P}}+\frac{Glc6P}{k_{m}^{Glc6P}}}$$

$k_{eq}^{G1PI}=$16.2 [[61](#_ENREF_61)]

$k_{m}^{Glc1P}=0.045$ mM [[60](#_ENREF_60)]

$k_{m}^{Glc6P}=0.67mM$ [[60](#_ENREF_60)]

## G6PT_ER_ (Glucose-6-phosphate transport to ER)

$${Glc6P}_{ER}\leftrightarrow Glc6P$$

$$v_{{G6PT}_{ER}}=V_{max}^{{G6PT}_{ER}}\cdot\frac{\left( Glc6P-{Glc6P}_{ER} \right)}{1+\frac{Glc6P}{k_{m}^{Glc6P}}+\frac{Glc6P_{ER}}{k_{m}^{Glc6P_{ER}}}}$$

$k_{m}^{{Glc6P}_{ER}}=1.12 mM$ [[50](#_ENREF_50)]

$k_{m}^{Glc6P}=1.12 mM$ [[50](#_ENREF_50)]

## GS (Glycogen synthase)

$$UDP\text{-}Glc\to UDP+Glyc$$

$$v_{GS}=V_{max}^{GS}\cdot\left( \left( 1-\gamma^{GS} \right)\cdot v_{GS}^{native}+\gamma^{GS}\cdot v_{GS}^{phospho} \right)$$

$$V_{max}^{GS}=V_{0}^{GS}\cdot\frac{\left( store-glyc \right)}{\left( store-glyc \right)+0.1\cdot store}$$

$$store= 375 mM$$

$$v_{GS}^{native}=\frac{UDP\text{-}Glc}{{UDP\text{-}Glc+ K}_{m-native}^{UDP\text{-}Glc}}$$

$$K_{m_{native}}^{UDP\text{-}Glc}=K_{0_{native}}^{UDP\text{-}Glc}\cdot\left( 1-\frac{Glc6P}{Glc6P+K_{a_{native}}^{Glc6p}} \right)+K_{b_{native}}^{UDP\text{-}Glc}$$

$K_{0_{native}}^{UDP\text{-}Glc}=$1.4 mM [[62](#_ENREF_62)]

$K_{a_{native}}^{Glc6P}=0.007$ mM [[62](#_ENREF_62)]

$K_{b_{native}}^{UDP\text{-}Glc}=0.2$ mM [[62](#_ENREF_62)]

$$v_{GS}^{phospho}=\frac{UDP\text{-}Glc}{{UDP\text{-}Glc+ K}_{m_{phospho}}^{UDP\text{-}Glc}}$$

$$k_{m_{phospho}}^{UDP\text{-}Glc}=K_{0_{phospho}}^{UDP\text{-}Glc}\cdot\left( 1-\frac{Glc6P}{Glc6P+K_{a_{phospho}}^{Glc6P}} \right)+K_{b_{phospho}}^{UDP\text{-}Glc}$$

$K_{0_{phospho}}^{UDP\text{-}Glc}=$ 32 mM [[62](#_ENREF_62)]

$K_{a_{phospho}}^{Glc6P}=0.09$ mM [[62](#_ENREF_62)]

$K_{b_{phospho}}^{UDP\text{-}Glc}=0.3$ mM [[62](#_ENREF_62)]

## HK (Hexokinase)

$$Glc+ATP\to Glc6P+ADP$$

$$v_{HK}=V_{max}^{HK}\cdot\frac{ATP}{{ATP+k}_{m}^{ATP}}\cdot\frac{Glc}{{Glc+k}_{m}^{Glc}}$$

$$k_{m}^{Glc}=k_{0}^{Glc}\cdot(1+\frac{Glc6P}{k_{i-Glc}^{Glc6P}})$$

$$k_{m}^{ATP}=k_{0}^{ATP}\cdot(1+\frac{Glc6P}{k_{i-ATP}^{Glc6P}})$$

$k_{0}^{Glc}=$0.42 mM {Kogure, 1996 #200}

$k_{i-Glc}^{Glc6p}=0.5 mM$ {Kogure, 1996 #200}

$k_{0}^{ATP}=2.09 mM$ {Kogure, 1996 #200}

$k_{i-ATP}^{Glc6P}=$ 0.19 mM {Kogure, 1996 #200}

## LacT (Lactate transporter)

$$Lac_{ext}\leftrightarrow Lac$$

$$v_{LacT}=v_{max}^{LacT}\cdot\frac{Lac_{ext}-Lac}{1+\frac{Lac}{k_{m}^{Lac}}+\frac{Lac_{ext}}{k_{m}^{Lac_{ext}}}}$$

$k_{m}^{Lac}=2.42 mM$ [[63](#_ENREF_63)]

$k_{m}^{Lac_{ext}}=2.42 mM$ [[63](#_ENREF_63)]

## LDH (Lactate dehydrogenase)

$$Pyr+NADH\leftrightarrow Lac+NAD^{+}$$

$$v_{LDH}=v_{max}^{LDH}*\frac{Pyr\cdot NADH-{Lac\cdot NAD^{+}}/{k_{eq}^{LDH}}}{\left( 1+\frac{NADH}{k_{m}^{NADH}} \right)\cdot\left( 1+\frac{Pyr}{k_{m}^{Pyr}} \right)+\left( 1+\frac{Lac}{k_{m}^{Lac}} \right)\cdot\left( 1+\frac{NAD^{+}}{k_{m}^{NAD^{+}}} \right)-1}$$

$k_{eq}^{LDH}=$9000 [[66](#_ENREF_66)]

$k_{m}^{NADH}=$ 0.015 mM [[65](#_ENREF_65)]

$k_{m}^{Pyr}=0.15 mM$ [[65](#_ENREF_65)]

$k_{m}^{Lac}=36 mM$ [[64](#_ENREF_64)]

$k_{m}^{NAD^{+}}=$0.11 mM [[65](#_ENREF_65)]

## MalT (Malate transporter)

$$Mal_{mito}+P\leftrightarrow Mal+P_{mito}$$

$$v_{MalT}=v_{max}^{MalT}\cdot\left( \frac{Mal_{mito}\cdot P-Mal\cdot P_{mito}}{\left( 1+\frac{Mal_{mito}}{K_{m}^{Mal_{mito}}} \right)\cdot\left( 1+\frac{P}{K_{m}^{P}} \right)+\left( 1+\frac{Mal}{K_{m}^{Mal}} \right)\cdot\left( 1+\frac{P_{mito}}{K_{m}^{P_{mito}}} \right)-1} \right)$$

$k_{m}^{P}=$1.41 mM [[67](#_ENREF_67)]

$k_{m}^{Mal_{mito}}=$ 0.49 mM [[67](#_ENREF_67)]

$k_{m}^{P_{mito}}=$1.41 mM [[67](#_ENREF_67)]

$k_{m}^{Mal}=$ 0.49 mM [[67](#_ENREF_67)]

## MDH (Malate dehydrogenase)

$$Mal+NAD^{+}\leftrightarrow OA+NADH$$

$$v_{MDH}=v_{max}^{MDH}\cdot\frac{Mal\cdot NAD^{+}-{OA\cdot NADH}/{k_{eq}^{MDH}}}{\left( 1+\frac{Mal}{k_{m}^{Mal}} \right)\cdot\left( 1+\frac{NAD^{+}}{k_{m}^{NAD^{+}}} \right)+\left( 1+\frac{OA}{k_{m}^{OA}} \right)\cdot\left( 1+\frac{NADH}{k_{m}^{NADH}} \right)-1}$$

$k_{eq}^{MDH}={3\cdot10}^{-5}$ [[68](#_ENREF_68)]

$k_{m}^{Mal}=1.1 mM$ [[69](#_ENREF_69)]

$k_{m}^{NAD^{+}}=0.114 mM$ [[69](#_ENREF_69)]

$k_{m}^{OA}=0.088 mM$ [[69](#_ENREF_69)]

$k_{m}^{NADH}=0.026 mM$ [[69](#_ENREF_69)]

## MDH_mito_ (Mitochondrial malate dehydrogenase)

$$Mal_{mito}+NAD_{mito}^{+}\leftrightarrow OA_{mito}+NADH_{mito}$$

$$v_{MDH_{mito}}=V_{max}^{MDH_{mito}}\cdot\left( \frac{{Mal}_{mito}\cdot NAD_{mito}^{+}-\frac{1}{K_{eq}^{MDH_{mito}}}\cdot OA_{mito}\cdot NADH_{mito}}{\left( 1+\frac{Mal_{mito}}{K_{m}^{Mal_{mito}}} \right)\cdot\left( 1+\frac{NAD_{mito}}{K_{m}^{NAD_{mito}}} \right)+\left( 1+\frac{OA_{mito}}{K_{m}^{OA_{mito}}} \right)\cdot\left( 1+\frac{NADH_{mito}}{K_{m}^{NADH_{mito}}} \right)-1} \right)$$

$K_{eq}^{MDH_{mito}}=3.1\cdot{10}^{-5}$ (pH 7.5) [[68](#_ENREF_68)]

$K_{m}^{Mal_{mito}}=$0.33 mM [[70](#_ENREF_70)]

$K_{m}^{{NAD}_{mito}}=$ 0.06 mM [[71](#_ENREF_71)]

$K_{m}^{{OA}_{mito}}=$ 0.017 mM [[71](#_ENREF_71)]

$K_{m}^{{NADH}_{mito}}=$0.044 mM [[71](#_ENREF_71)]

## NDK^GTP^, NDK^UTP^, NDK^GTP^_mito_ (Cytosolic and mitochondrial nucleoside-diphosphate kinases)

$$ATP_{mito}+GDP_{mito}\leftrightarrow ADP_{mito}+GTP_{mito}$$

$$ATP+GDP\leftrightarrow ADP+GTP$$

$$ATP+UDP\leftrightarrow ADP+UTP$$

$$v_{NDK^{GTP}}=v_{max}^{NDK^{GTP}}\cdot\frac{ATP\cdot GDP-{ADP\cdot GTP}/{k_{eq}^{NDK}}}{\left( 1+\frac{ATP}{k_{m}^{ATP}} \right)\left( 1+\frac{GDP}{k_{m}^{GDP}} \right)+\left( 1+\frac{ADP}{k_{m}^{ADP}} \right)\left( 1+\frac{GTP}{k_{m}^{GTP}} \right)-1}$$

$$v_{NDK^{UTP}}=v_{max}^{NDK^{UTP}}\cdot\frac{ATP\cdot UDP-{ADP\cdot UTP}/{k_{eq}^{NDK}}}{\left( 1+\frac{ATP}{k_{m}^{ATP}} \right)\left( 1+\frac{UDP}{k_{m}^{UDP}} \right)+\left( 1+\frac{ADP}{k_{m}^{ADP}} \right)\left( 1+\frac{UTP}{k_{m}^{UTP}} \right)-1}$$

$$v_{{NDK^{GTP}}_{mito}}=v_{max}^{{Ndk^{GTP}}_{mito}}\cdot\frac{ATP_{mito}\cdot GDP_{mito}-{ADP_{mito}\cdot GTP_{mito}}/{k_{eq}^{NDK}}}{\left( 1+\frac{ATP_{mito}}{k_{m}^{ATP_{mito}}} \right)\left( 1+\frac{GDP_{mito}}{k_{m}^{GDP_{mito}}} \right)+\left( 1+\frac{ADP_{mito}}{k_{m}^{ADP_{mito}}} \right)\left( 1+\frac{GTP_{mito}}{k_{m}^{GTP_{mito}}} \right)-1}$$

$k_{eq}^{NDK}=1$ [[72](#_ENREF_72)]

$k_{m}^{ATP}=1.33 mM$ [[73](#_ENREF_73)]

$k_{m}^{GDP}=3.1*{10}^{-2} mM$[[73](#_ENREF_73)]

$k_{m}^{ADP}=4.2*{10}^{-2}mM$ [[73](#_ENREF_73)]

$k_{m}^{GTP}=0.15 mM$ [[74](#_ENREF_74)]

$K_{m}^{ATP_{mito}}=$ 1.66 mM [[73](#_ENREF_73)]

$K_{m}^{GDP_{mito}}=$0.036 mM [[73](#_ENREF_73)]

$K_{m}^{ADP_{mito}}=$0.073 mM [[73](#_ENREF_73)]

$K_{m}^{GTP\_mito}=0.15$ mM [[74](#_ENREF_74)]

$k_{m}^{UTP}=16 mM$[[74](#_ENREF_74)]

$k_{m}^{UDP}=0.19 mM$[[73](#_ENREF_73)]

## PC (Pyruvate carboxylase)

$$ATP_{mito}+Pyr_{mito}+C{O_{2}}_{mito}\leftrightarrow OA_{mito}+ADP_{mito}+P_{mito}$$

$$v_{PC}=v_{max}^{PC}\cdot\frac{ATP_{mito}\cdot Pyr_{mito}\cdot CO_{2_{mito}}-{OA_{mito}\cdot ADP_{mito}\cdot P_{mito}}/{k_{eq}^{PC}}}{\left( ATP_{mito}+k_{m}^{ATP_{mito}} \right)\cdot\left( Pyr_{mito}+k_{m}^{Pyr_{mito}} \right)\cdot\left( C{O_{2}}_{mito}+k_{m}^{C{O_{2}}_{mito}} \right)}$$

$k_{m}^{ATP_{mito}}=$0.14 mM [[75](#_ENREF_75)]

$k_{m}^{Pyr_{mito}}=$0.33 mM [[75](#_ENREF_75)]

$k_{m}^{C{O_{2}}_{mito}}=$4.2 mM [[75](#_ENREF_75)]

$k_{eq}^{PC}= 6.55$ [[76](#_ENREF_76)]

## PEPCK (Phosphoenolpyruvate carboxykinase)

$$OA+GTP\leftrightarrow PEP+GDP+CO_{2}$$

$$v_{PEPCK}=v_{max}^{PEPCK}\cdot\frac{OA\cdot GTP-{PEP\cdot GDP*CO_{2}}/{k_{eq}^{PEPCK}}}{\left( 1+\frac{OA}{k_{m}^{OA}} \right)\cdot\left( 1+\frac{GTP}{k_{m}^{GTP}} \right)+\left( 1+\frac{PEP}{k_{m}^{PEP}} \right)\cdot\left( 1+\frac{GDP}{k_{m}^{GDP}} \right)\cdot\left( 1+\frac{CO_{2}}{k_{m}^{CO_{2}}} \right)-1}$$

$k_{eq}^{PEPCK}=$110 mM [[77](#_ENREF_77)]

$k_{m}^{OA}=$ 0.024 mM [[78](#_ENREF_78)]

$k_{m}^{GTP}=$0.021 mM [[79](#_ENREF_79)]

$k_{m}^{PEP}=0.4 mM$ [[80](#_ENREF_80)]

$k_{m}^{GDP}=$0.02 mM [[81](#_ENREF_81)]

$k_{m}^{CO_{2}}=$1.194 mM [[82](#_ENREF_82)]

## PEPCK_mito_ (Mitochondrial phosphoenolpyruvate carboxykinase)

$$OA_{mito}+GTP_{mito}\leftrightarrow PEP_{mito}+GDP_{mito}+CO_{2_{mito}}$$

$$v_{PEPCK_{mito}}=v_{max}^{PEPCK_{mito}}\cdot\frac{OA_{mito}\cdot GTP_{mito}-{PEP_{mito}\cdot GDP_{mito}\cdot CO_{2_{mito}}}/{k_{eq}^{PEPCK_{mito}}}}{\left( 1+\frac{OA_{mito}}{k_{m^{mito}}^{OA}} \right)\cdot\left( 1+\frac{GTP_{mito}}{k_{m^{mito}}^{GTP}} \right)+\left( 1+\frac{P{EP}_{mito}}{k_{m}^{PEP_{mito}}} \right)\cdot\left( 1+\frac{GDP_{mito}}{k_{m}^{GDP_{mito}}} \right)\cdot\left( 1+\frac{CO_{2_{mito}}}{k_{m}^{CO_{2_{mito}}}} \right)-1}$$

$k_{eq}^{PEPCK_{mito}}=$160 mM [[77](#_ENREF_77)]

$k_{m}^{OA_{mito}}=$ 0.0085 mM [[83](#_ENREF_83)]

$k_{m}^{GTP_{mito}}=$0.022 mM [[81](#_ENREF_81)]

$k_{m}^{PEP_{mito}}=0.4 mM$ [[80](#_ENREF_80)]

$k_{m}^{GDP_{mito}}=$0.02 mM [[81](#_ENREF_81)]

$k_{m}^{CO_{2_{mito}}}=$1.06 mM [[84](#_ENREF_84)]

## PEPT (Phosphoenolpyruvate transporter)

$$PEP\leftrightarrow PEP_{mito}$$

$$v_{PEPT}=v_{max}^{PEPT}\cdot\frac{PEP_{mito}-{PEP}/{k_{eq}^{PEPT}}}{1+\frac{PEP}{k_{m}^{PEP}}+\frac{PEP_{mito}}{k_{m}^{PEP_{mito}}}}$$

$$k_{eq}^{PEPT}=\exp\left( -\frac{Vmm\cdot F}{R\cdot T} \right)$$

$k_{m}^{PEP}=0.1 mM$ [[85](#_ENREF_85)]

$k_{m}^{PEP_{mito}}=0.1 mM$ [[85](#_ENREF_85)]

## PFK1 (Phosphofructokinase 1)

$$Fru6P+ATP\to Fru16P+ADP$$

$$v_{PFK1}=v_{max}^{PFK1}\cdot\frac{ATP}{ATP+K_{m}^{ATP}}\cdot\left( 1-\frac{ATP^{n_{i}}}{ATP^{n_{i}}+\left( K_{i}^{ATP} \right)^{n_{i}}} \right)\cdot\frac{\left( Fru6P \right)^{n_{Fru6P}}}{\left( Fru6P \right)^{n_{Fru6P}}+\left( k_{m}^{Fru6P} \right)^{n_{Fru6P}}}$$

$K_{m}^{ATP}=K_{0}^{ATP}\cdot\left( 1-\frac{Fru26P_{2}}{Fru26P_{2} +K_{a}^{Fru26P_{2}}} \right)$

$K_{0}^{ATP}=$0.2 mM [[86](#_ENREF_86), [87](#_ENREF_87)]

$K_{a}^{Fru26P_{2}}=0.0027$ mM [[86](#_ENREF_86), [87](#_ENREF_87)]

$$K_{i}^{ATP}=K_{i0}^{ATP}\cdot\left( {1+ f}_{Fru26P_{2}}\frac{Fru26P_{2}}{Fru26P_{2} +K_{a2}^{Fru26P_{2}}} \right)$$

$K_{i0}^{ATP}=$ 0.7 mM [[86](#_ENREF_86), [87](#_ENREF_87)]

$f_{Fru26P_{2}}=9$ [[86](#_ENREF_86), [87](#_ENREF_87)]

$K_{a2}^{Fru26P_{2}}=0.54 mM$ [[86](#_ENREF_86), [87](#_ENREF_87)]

$n_{i}=$4 [[86](#_ENREF_86), [87](#_ENREF_87)]

$$k_{m}^{Fru6P}=K_{0}^{Fru6p}\cdot\left( 1+\frac{ATP}{k_{i}^{ATP}} \right)\cdot\left( 1+\frac{Cit}{k_{i}^{Cit}} \right)\cdot\left( 1-f_{AMP}\frac{AMP^{n_{AMP}}}{AMP^{n_{AMP}}+\left( K_{a}^{AMP} \right)^{n_{AMP}}} \right)\cdot\left( 1-f_{P}\frac{P}{P+K_{a}^{P}} \right)\cdot\left( 1-f_{Fru26P_{2}}\frac{{Fru26P}_{2}^{n_{Fru26P_{2}}}}{{Fru26P}_{2}^{n_{Fru26P_{2}}}+\left( K_{a}^{Fru26P_{2}} \right)^{n_{Fru26P_{2}}}} \right)$$

$K_{0}^{Fru6P}=1.14$ mM [[88](#_ENREF_88)]

$k_{i}^{ATP}=0.6$ mM [[88](#_ENREF_88)]

$k_{i}^{Cit}=3.27$ mM [[88](#_ENREF_88)]

$f_{AMP}=0.77$ [[88](#_ENREF_88)]

$K_{a}^{AMP}=0.1$ mM [[88](#_ENREF_88)]

$n_{AMP}=1.84$ [[88](#_ENREF_88)]

$f_{P}=0.85$ [[88](#_ENREF_88)]

$K_{a}^{P}=0.69$ mM [[88](#_ENREF_88)]

$f_{Fru26P_{2}}=0.92$ [[86](#_ENREF_86)]

$K_{a}^{Fru26P_{2}}=0.0045 mM$ [[86](#_ENREF_86)]

$n_{Fru26P_{2}}=1.2$ [[86](#_ENREF_86)]

$$n^{Fru6P}=\left( n_{0}+\frac{ATP^{n_{ATP}}}{ATP^{n_{ATP}}+\left( K_{i}^{ATP} \right)^{n_{ATP}}} \right)\cdot\left( 1-f_{AMP}\frac{AMP^{n_{AMP}}}{AMP^{n_{AMP}}+\left( K_{a}^{AMP} \right)^{n_{AMP}}} \right)\cdot\left( 1+f_{Cit}\frac{Cit^{n_{Cit}}}{Cit^{n_{Cit}}+\left( K_{i}^{Cit} \right)^{n_{Cit}}} \right)\cdot\left( 1-f_{P}\frac{P^{n_{P}}}{P^{n_{P}}+\left( K_{a}^{P} \right)^{n_{P}}} \right)\cdot\cdot\left( 1-f_{Fru26P_{2}}\frac{{Fru26P_{2}}^{n_{Fru26bp}}}{{Fru26P}_{2}^{n_{Fru26P_{2}}}+\left( K_{a}^{Fru26P_{2}} \right)^{n_{Fru26P_{2}}}} \right)$$

$n_{0}=3.67$ [[88](#_ENREF_88)]

$K_{i}^{ATP}=0.13$ mM [[88](#_ENREF_88)]

$n_{ATP}=1.59$ [[88](#_ENREF_88)]

$f_{AMP}=0.4$ [[88](#_ENREF_88)]

$K_{a}^{AMP}=0.086$ mM [[88](#_ENREF_88)]

$n_{AMP}=2.22$ [[88](#_ENREF_88)]

$f_{Cit}=0.1$ [[88](#_ENREF_88)]

$K_{i}^{Cit}=0.18$ mM [[88](#_ENREF_88)]

$n_{Cit}=4$ [[88](#_ENREF_88)]

$f_{P}=0.28$ [[88](#_ENREF_88)]

$K_{a}^{P}=0.53$mM [[88](#_ENREF_88)]

$n_{P}=4$ [[88](#_ENREF_88)]

$f_{Fru26P_{2}}=0.37$ [[86](#_ENREF_86)]

$K_{a}^{Fru26P_{2}}=0.0021$mM [[86](#_ENREF_86)]

$n^{Fru26P_{2}}=4$ [[86](#_ENREF_86)]

## PFK2/FBP2 (Phosphofructokinase 2/Fructose-2,6-bisphosphatase)

$$Fru6P+ATP\to Fru26P_{2}+ADP$$

$$v_{PFK2}=\left( 1-\gamma^{PFK2} \right)\cdot v_{PFK2}^{native}+\gamma^{PFK2}\cdot v_{PFK2}^{phospho}$$

$$v_{PFK2}^{native}=V_{max}^{PFK2}\frac{Fru6P^{n}}{Fru6P^{n}+\left( k_{m^{native}}^{Fru6P} \right)^{n}}\cdot\frac{ATP}{{ATP+k}_{m}^{ATP}}\cdot\left( 1-n_{0}\cdot\frac{PEP}{PEP+k_{i}^{PEP}} \right)$$

$k_{m^{native}}^{Fru6P}=0.015$mM [[89](#_ENREF_89)]

$n=$1.3 [[89](#_ENREF_89)]

$k_{m}^{ATP}=0.25 mM$ [[89](#_ENREF_89)]

$k_{i}^{PEP}=0.25 mM$ [[90](#_ENREF_90)]

$n_{0}=0.85$ [[90](#_ENREF_90)]

$$v_{PFK2}^{phospho}=V_{max}^{PFK2}\frac{Fru6P^{n}}{{{Fru6P^{n}+k}_{m^{phospho}}^{Fru6P}}^{n}}\cdot\frac{ATP}{{ATP+k}_{m}^{ATP}}\cdot\left( 1-n_{0}\cdot\frac{PEP}{PEP+k_{i}^{PEP}} \right)$$

$k_{m^{phospho}}^{Fru6P}=$ 0.05 mM [[89](#_ENREF_89)]

$n=$2 [[89](#_ENREF_89)]

$k_{m}^{ATP}=0.5 mM$ [[89](#_ENREF_89)]

$k_{i}^{PEP}=0.25 mM$ [[90](#_ENREF_90)]

$n_{0}=0.85$ [[90](#_ENREF_90)]

$$Fru26P_{2}\to Fru6P+P$$

$$v_{FBP2}=V_{max}^{FBP2}*\left( \left( 1-\gamma^{FBP2} \right)\cdot v_{FBP2}^{native}+\gamma^{FBP2}\cdot v_{FBP2}^{phospho} \right)$$

$$v_{FBP2}^{native}=\frac{Fru26P_{2}}{{Fru26P_{2}+k}_{m^{native}}^{Fru26P_{2}}}/\left( 1+\frac{Fru6P}{k_{i^{native}}^{Fru6P}} \right)$$

$k_{m^{native}}^{Fru26P_{2}}=0.01 mM$ [[91](#_ENREF_91)]

$k_{i^{native}}^{Fru6P}=0.0035$ mM [[89](#_ENREF_89)]

$$v_{FBP2}^{phospho}=\frac{Fru26P_{2}}{{Fru26P_{2}+k}_{m^{phospho}}^{Fru26P_{2}}}/\left( 1+\frac{Fru6P}{k_{i^{phospho}}^{Fru6P}} \right)$$

$k_{m^{phospho}}^{Fru26P_{2}}=0.0005$mM [[89](#_ENREF_89)]

$k_{i^{phospho}}^{Fru6P}=0.01 mM$ [[89](#_ENREF_89)]

## PGK (Phosphoglycerate kinase)

$$ADP+13P2G\to ATP+3PG$$

$$v_{PGK}=v_{max}^{PGK}*\frac{ADP\cdot13P2G-{ATP\cdot3PG}/{k_{eq}^{PGK}}}{\left( 1+\frac{ADP}{k_{m}^{ADP}} \right)\cdot\left( 1+\frac{13P2G}{k_{m}^{13P2G}} \right)+\left( 1+\frac{ATP}{k_{m}^{ATP}} \right)\cdot\left( 1+\frac{3PG}{k_{m}^{3PG}} \right)-1}$$

$k_{eq}^{PGK}= 1830$[[92](#_ENREF_92)]

$k_{m}^{ADP}=0.35 mM$ [[93](#_ENREF_93)]

$k_{m}^{13P2G}=0.0022 mM$ [[93](#_ENREF_93)]

$k_{m}^{ATP}=0.24 mM$ [[94](#_ENREF_94)]

$k_{m}^{3PG}=1.65 mM$ [[94](#_ENREF_94)]

## PGM (Phosphoglycerate mutase)

$$3PG\leftrightarrow2PG$$

$$v_{PGM}=v_{max}^{PGM}\cdot\frac{3PG-{2PG}/{k_{eq}^{PGM}}}{1+\frac{3PG}{k_{m}^{3PG}}+\frac{2PG}{K_{m}^{2PG}}}$$

$k_{eq}^{PGM}=$0.096 [[95](#_ENREF_95)]

$k_{m}^{3PG}=0.52 mM$ [[96](#_ENREF_96)]

$K_{m}^{2PG}=0.24 mM$ [[96](#_ENREF_96)]

## PK (Pyruvate kinase)

$$PEP+ADP\leftrightarrow Pyr+ATP$$

$$v_{PK}=v_{max}^{PK}\cdot\left( \left( 1-\gamma^{PK} \right)\cdot v_{PK}^{native}+\gamma^{PK}\cdot v_{PK}^{phospho} \right)$$

$$v_{PK}^{native}=\frac{PEP}{{PEP+k}_{m^{native}}^{PEP}\cdot\left( 1+\frac{ATP}{k_{i^{native}}^{ATP}} \right)\cdot\left( 1-\frac{Fru16P_{2}}{Fru16P_{2}+k_{a^{native}}^{Fru16P_{2}}} \right)}\cdot\frac{ADP}{{ADP+k}_{m}^{ADP}}$$

$k_{m^{native}}^{PEP}=$0.13 mM [[97](#_ENREF_97)]

$k_{i^{native}}^{ATP}=$1 mM [[97](#_ENREF_97)]

$k_{a^{native}}^{Fru16P_{2}}=$0.0078 mM [[98](#_ENREF_98)]

$k_{m}^{ADP}=$ 0.25 mM [[99](#_ENREF_99)]

$$v_{PK}^{phospho}=\frac{PEP^{n}}{{PEP}^{n}+\left( k_{m^{phospho}}^{PEP}\cdot\left( 1+\frac{ATP}{k_{i^{phospho}}^{ATP}} \right)\cdot\left( 1-\frac{Fru16P_{2}}{Fru16P_{2}+k_{a^{phospho}}^{Fru16P_{2}}} \right) \right)^{n}}\cdot\frac{ADP}{{ADP+k}_{m}^{ADP}}$$

$k_{m^{phospho}}^{PEP}=$5.8 mM [[97](#_ENREF_97)]

$n=$2.9 [[97](#_ENREF_97)]

$k_{a^{phospho}}^{Fru16P_{2}}=$0.0095 mM [[98](#_ENREF_98)]

$k_{i^{phospho}}^{ATP}=0.32 mM$ [[32](#_ENREF_32)]

$k_{m}^{ADP}=$0.33 mM [[99](#_ENREF_99)]

## PyrMalT (Pyruvate/malate antiporter)

$$Mal_{mito}+Pyr\leftrightarrow Mal+{Pyr}_{mito}$$

$$v_{PyrMalT}=v_{max}^{PyrMalT}\cdot\left( \frac{Mal_{mito}\cdot Pyr-Mal\cdot{Pyr}_{mito}}{\left( 1+\frac{Mal_{mito}}{K_{m}^{Mal_{mito}}} \right)\cdot\left( 1+\frac{Pyr}{K_{m}^{Pyr}} \right)+\left( 1+\frac{Mal}{K_{m}^{Mal}} \right)\cdot\left( 1+\frac{Pyr_{mito}}{K_{m}^{Pyr_{mito}}} \right)-1} \right)$$

$k_{m}^{Pyr}=0.84 mM$ [[100](#_ENREF_100)]

$k_{m}^{Mal}=0.7 mM$ [[85](#_ENREF_85)]

$k_{m}^{Pyr_{mito}}=0.84 mM$ [[100](#_ENREF_100)]

$k_{m}^{Mal_{mito}}=0.7 mM$ [[85](#_ENREF_85)]

## PyrT (Pyruvate transporter)

$$Pyr\leftrightarrow Pyr_{mito}$$

$$v_{PyrT}=v_{max}^{PyrT}\cdot\frac{Pyr\cdot H^{+}-{Pyr_{mito}\cdot H}_{mito}^{+}}{1+\frac{Pyr}{k_{m}^{Pyr}}+\frac{Pyr_{mito}}{k_{m}^{Pyr_{mito}}}}$$

$k_{m}^{Pyr}=$0.15 mM [[101](#_ENREF_101)]

$k_{m}^{Pyr_{mito}}=$0.15 mM [[101](#_ENREF_101)]

## TPI (Triosephosphate isomerase)

$$DHAP\leftrightarrow GAP$$

$$v_{TPI}=v_{max}^{TPI}\cdot\frac{DHAP-{GAP}/{k_{eq}^{TPI}}}{1+\frac{DHAP}{k_{m}^{DHAP}}+\frac{GAP}{k_{m}^{GAP}}}$$

$k_{eq}^{TPI}=$0.04545 [[37](#_ENREF_37)]

$k_{m}^{DHAP}=$0.59 mM [[102](#_ENREF_102)]

$k_{m}^{GAP}=$0.415 mM [[102](#_ENREF_102)]

## UGT (Uridine diphospho-glucuronosyltransferase)

$$UTP+Glc1P\leftrightarrow UDP\text{-}Glc+PP$$

$$v_{UGT}=v_{max}^{UGT}*\frac{UTP*Glc1P-{UDP\text{-}Glc*PP}/{k_{eq}^{UGT}}}{\left( 1+\frac{UTP}{k_{m}^{UTP}} \right)\left( 1+\frac{Glc1P}{k_{m}^{Glc1P}} \right)+\left( 1+\frac{UDP\text{-}Glc}{k_{m}^{UDP\text{-}Glc}} \right)\left( 1+\frac{PP}{k_{m}^{PP}} \right)-1}$$

$k_{eq}^{UGT}=0.3122$[[103](#_ENREF_103)]

$k_{m}^{UTP}=$0.2 mM [[103](#_ENREF_103)]

$k_{m}^{Glc1P}=$0.055 mM [[103](#_ENREF_103)]

$k_{m}^{UDP\text{-}Glc}=$0.06 mM [[103](#_ENREF_103)]

$k_{m}^{PP}=$0.084 mM [[103](#_ENREF_103)]

# Vmax values

| Reaction | Parameter | Dimension | Periportal | Intermediate | Pericentral |
| --- | --- | --- | --- | --- | --- |
| ALD | $v_{max}^{ALD}$ | $h^{-1}$ | 7.78E+08 | 7.78E+08 | 7.78E+08 |
| EN | $v_{max}^{EN}$ | $h^{-1}$ | 1.94E+10 | 1.94E+10 | 1.94E+10 |
| FBP1 | $V_{max}^{FBP1}$ | $mM\cdot h^{-1}$ | 4.04E+04 | 2.92E+04 | 1.80E+04 |
| GAPDH | $v_{max}^{GAPDH}$ | $h^{-1}\cdot mM^{-2}$ | 2.92E+08 | 2.92E+08 | 2.92E+08 |
| GK | $V_{0}^{GK}$ | ${mM h}^{-1}$ | 3.18E+03 | 4.77E+03 | 6.36E+03 |
| GlcT_ER_ | $V_{max}^{{Glc}_{ER}}$ | $h^{-1}$ | 1.94E+10 | 1.94E+10 | 1.94E+10 |
| GLUT2 | $V_{max}^{GLUT2}$ | $h^{-1}$ | 9.00E+01 | 9.00E+01 | 9.00E+01 |
| GP | $V_{0}^{GP}$ | $mM\cdot h^{-1}$ | 1.93E+02 | 1.40E+02 | 8.69E+01 |
| G6P_ER_ | $V_{max}^{{G6P}_{ER}}$ | $mM\cdot h^{-1}$ | 8.91E+02 | 6.48E+02 | 4.05E+02 |
| GPI | $V_{max}^{GPI}$ | $h^{-1}$ | 1.07E+09 | 1.07E+09 | 1.07E+09 |
| G1PI | $v_{max}^{G1PI}$ | $h^{-1}$ | 6.48E+07 | 6.48E+07 | 6.48E+07 |
| G6PT_ER_ | $V_{max}^{{G6PT}_{ER}}$ | $h^{-1}$ | 1.94E+10 | 1.94E+10 | 1.94E+10 |
| GS | $V_{0}^{GS}$ | $mM\cdot h^{-1}$ | 1.02E+02 | 6.80E+01 | 3.45E+01 |
| HK | $V_{max}^{HK}$ | $mM\cdot h^{-1}$ | 1.17E+02 | 1.17E+02 | 1.17E+02 |
| LacT | $v_{max}^{LacT}$ | $h^{-1}$ | 5.83E+02 | 5.83E+02 | 5.83E+02 |
| LDH | $v_{max}^{LDH}$ | $h^{-1}\cdot mM^{-1}$ | 1.56E+11 | 1.56E+11 | 1.56E+11 |
| MalT | $v_{max}^{MalT}$ | $h^{-1}\cdot mM^{-1}$ | 1.94E+03 | 1.94E+03 | 1.94E+03 |
| MDH | $v_{max}^{MDH}$ | $h^{-1}\cdot mM^{-1}$ | 1.94E+09 | 1.94E+09 | 1.94E+09 |
| MDH_mito_ | $V_{max}^{MDH_{mito}}$ | $h^{-1}\cdot mM^{-1}$ | 6.80E+11 | 6.80E+11 | 6.80E+11 |
| NDK^GTP^ | $v_{max}^{NDK^{GTP}}$ | $h^{-1}\cdot mM^{-1}$ | 1.94E+11 | 1.94E+11 | 1.94E+11 |
| NDK^UTP^ | $v_{max}^{NDK^{UTP}}$ | $h^{-1}\cdot mM^{-1}$ | 1.62E+07 | 1.62E+07 | 1.62E+07 |
| NDK^GTP^_mito_ | $v_{max}^{NDK^{GTP}mito}$ | $h^{-1}\cdot mM^{-1}$ | 1.94E+07 | 1.94E+07 | 1.94E+07 |
| PC | $v_{max}^{PC}$ | $mM\cdot h^{-1}$ | 4.46E+03 | 4.46E+03 | 4.46E+03 |
| PEPCK | $v_{max}^{PEPCK}$ | $h^{-1}\cdot mM^{-1}$ | 1.09E+07 | 7.18E+06 | 3.42E+06 |
| PEPCK_mito_ | $v_{max}^{PEPCK_{mito}}$ | $h^{-1}\cdot mM^{-1}$ | 1.98E+06 | 1.98E+06 | 1.98E+06 |
| PEPT | $v_{max}^{PEPT}$ | $h^{-1}$ | 1.94E+05 | 1.94E+05 | 1.94E+05 |
| PFK1 | $v_{max}^{PFK1}$ | $mM\cdot h^{-1}$ | 5.46E+04 | 5.76E+04 | 6.06E+04 |
| PFK2/FBP2 | $V_{max}^{PFK2}$ | $mM\cdot h^{-1}$ | 6.64E+01 | 9.97E+01 | 1.33E+02 |
| PFK2/FBP2 | $V_{max}^{FBP2}$ | $mM\cdot h^{-1}$ | 3.58E+02 | 5.37E+02 | 7.15E+02 |
| PGK | $v_{max}^{PGK}$ | $h^{-1}\cdot mM^{-1}$ | 1.94E+10 | 1.94E+10 | 1.94E+10 |
| PGM | $v_{max}^{PGM}$ | $h^{-1}$ | 1.94E+10 | 1.94E+10 | 1.94E+10 |
| PK | $v_{max}^{PK}$ | $mM\cdot h^{-1}$ | 6.13E+03 | 7.45E+03 | 8.77E+03 |
| PyrMalT | $v_{max}^{PyrMalT}$ | $h^{-1}\cdot mM^{-1}$ | 1.94E+04 | 1.94E+04 | 1.94E+04 |
| PyrT | $v_{max}^{PyrT}$ | $h^{-1}\cdot mM^{-1}$ | 1.94E+08 | 1.94E+08 | 1.94E+08 |
| TPI | $v_{max}^{TPI}$ | $h^{-1}$ | 1.94E+08 | 1.94E+08 | 1.94E+08 |
| UGT | $v_{max}^{UGT}$ | $h^{-1}\cdot mM^{-1}$ | 6.48E+09 | 6.48E+09 | 6.48E+09 |

# References

1. Tischler ME, Hecht P, Williamson JR: Determination of mitochondrial/cytosolic metabolite gradients in isolated rat liver cells by cell disruption. Archives of biochemistry and biophysics 1977, 181(1):278-293.

2. Siess EA, Brocks DG, Lattke HK, Wieland OH: Effect of glucagon on metabolite compartmentation in isolated rat liver cells during gluconeogenesis from lactate. Biochem J 1977, 166(2):225-235.

3. Albe KR, Butler MH, Wright BE: Cellular concentrations of enzymes and their substrates. Journal of theoretical biology 1990, 143(2):163-195.

4. Start C, Newsholme EA: The effects of starvation and alloxan-diabetes on the contents of citrate and other metabolic intermediates in rat liver. Biochem J 1968, 107(3):411-415.

5. Konig M, Bulik S, Holzhutter HG: Quantifying the contribution of the liver to glucose homeostasis: a detailed kinetic model of human hepatic glucose metabolism. PLoS Comput Biol 2012, 8(6):e1002577.

6. Jackson RC, Lui MS, Boritzki TJ, Morris HP, Weber G: Purine and pyrimidine nucleotide patterns of normal, differentiating, and regenerating liver and of hepatomas in rats. Cancer research 1980, 40(4):1286-1291.

7. Keppler DO, Pausch J, Decker K: Selective uridine triphosphate deficiency induced by D-galactosamine in liver and reversed by pyrimidine nucleotide precursors. Effect on ribonucleic acid synthesis. The Journal of biological chemistry 1974, 249(1):211-216.

8. Smith CM, Bryla J, Williamson JR: Regulation of mitochondrial alpha-ketoglutarate metabolism by product inhibition at alpha-ketoglutarate dehydrogenase. The Journal of biological chemistry 1974, 249(5):1497-1505.

9. Greenbaum AL, Gumaa KA, McLean P: The distribution of hepatic metabolites and the control of the pathways of carbohydrate metabolism in animals of different dietary and hormonal status. Archives of biochemistry and biophysics 1971, 143(2):617-663.

10. Lagunas R, McLean P, Greenbaum AL: The effect of raising the NAD+ content on the pathways of carbohydrate metabolism and lipogenesis in rat liver. Eur J Biochem 1970, 15(1):179-190.

11. Krebs HA: The redox state of nicotinamide adenine dinucleotide in the cytoplasm and mitochondria of rat liver. Advances in enzyme regulation 1967, 5:409-434.

12. Veech RL, Veloso D, Mehlman MA: Thiamin deficiency: liver metabolite levels and redox and phosphorylation states in thiamin-deficient rats. The Journal of nutrition 1973, 103(2):267-272.

13. Birt LM, Bartley W: The behaviour of pyridine nucleotides of mitochondria in a 'saline medium'. Biochem J 1960, 76:328-341.

14. Wilson DF, Stubbs M, Oshino N, Erecinska M: Thermodynamic relationships between the mitochondrial oxidation-reduction reactions and cellular ATP levels in ascites tumor cells and perfused rat liver. Biochemistry 1974, 13(26):5305-5311.

15. Aw TY, Andersson BS, Jones DP: Mitochondrial transmembrane ion distribution during anoxia. The American journal of physiology 1987, 252(4 Pt 1):C356-361.

16. Werkheiser WC, Bartley W: The study of steady-state concentrations of internal solutes of mitochondria by rapid centrifugal transfer to a fixation medium. Biochem J 1957, 66(1):79-91.

17. Guynn RW, Veloso D, Lawson JW, Veech RL: The concentration and control of cytoplasmic free inorganic pyrophosphate in rat liver in vivo. Biochem J 1974, 140(3):369-375.

18. Hitchings GH: Indications for control mechanisms in purine and pyrimidine biosynthesis as revealed by studies with inhibitors. Advances in enzyme regulation 1974, 12:121-129.

19. Keppler D, Frohlich J, Reutter W, Wieland O, Decker K: Changes in uridine nucleotides during liver perfusion with D-galactosamine. FEBS letters 1969, 4(4):278-280.

20. Mitchell P, Moyle J: Estimation of membrane potential and pH difference across the cristae membrane of rat liver mitochondria. Eur J Biochem 1969, 7(4):471-484.

21. la Fleur SE, Kalsbeek A, Wortel J, Fekkes ML, Buijs RM: A daily rhythm in glucose tolerance: a role for the suprachiasmatic nucleus. Diabetes 2001, 50(6):1237-1243.

22. Frangioudakis G, Gyte AC, Loxham SJ, Poucher SM: The intravenous glucose tolerance test in cannulated Wistar rats: a robust method for the in vivo assessment of glucose-stimulated insulin secretion. Journal of pharmacological and toxicological methods 2008, 57(2):106-113.

23. Hara E, Saito M: Diurnal changes in plasma glucose and insulin responses to oral glucose load in rats. The American journal of physiology 1980, 238(5):E463-466.

24. Balks HJ, Jungermann K: Regulation of peripheral insulin/glucagon levels by rat liver. Eur J Biochem 1984, 141(3):645-650.

25. Diaz B, Blazquez E: Effect of pinealectomy on plasma glucose, insulin and glucagon levels in the rat. Hormone and metabolic research = Hormon- und Stoffwechselforschung = Hormones et metabolisme 1986, 18(4):225-229.

26. Patel DG: Lack of glucagon response to hypoglycemia in long-term experimental diabetic rats. Diabetes 1983, 32(1):55-60.

27. Wan CK, Giacca A, Matsuhisa M, El-Bahrani B, Lam L, Rodgers C, Shi ZQ: Increased responses of glucagon and glucose production to hypoglycemia with intraperitoneal versus subcutaneous insulin treatment. Metabolism 2000, 49(8):984-989.

28. Zhou H, Tran PO, Yang S, Zhang T, LeRoy E, Oseid E, Robertson RP: Regulation of alpha-cell function by the beta-cell during hypoglycemia in Wistar rats: the "switch-off" hypothesis. Diabetes 2004, 53(6):1482-1487.

29. Bartrons R, Hue L, Van Schaftingen E, Hers HG: Hormonal control of fructose 2,6-bisphosphate concentration in isolated rat hepatocytes. Biochem J 1983, 214(3):829-837.

30. El-Maghrabi MR, Claus TH, Pilkis J, Fox E, Pilkis SJ: Regulation of rat liver fructose 2,6-bisphosphatase. The Journal of biological chemistry 1982, 257(13):7603-7607.

31. Pilkis S, Schlumpf J, Pilkis J, Claus TH: Regulation of phosphofructokinase activity by glucagon in isolated rat hepatocytes. Biochem Biophys Res Commun 1979, 88(3):960-967.

32. Feliu JE, Hue L, Hers HG: Hormonal control of pyruvate kinase activity and of gluconeogenesis in isolated hepatocytes. Proc Natl Acad Sci U S A 1976, 73(8):2762-2766.

33. Claus TH, El-Maghrabi MR, Pilkis SJ: Modulation of the phosphorylation state of rat liver pyruvate kinase by allosteric effectors and insulin. The Journal of biological chemistry 1979, 254(16):7855-7864.

34. Syed NA, Khandelwal RL: Reciprocal regulation of glycogen phosphorylase and glycogen synthase by insulin involving phosphatidylinositol-3 kinase and protein phosphatase-1 in HepG2 cells. Molecular and cellular biochemistry 2000, 211(1-2):123-136.

35. Hartmann H, Probst I, Jungermann K, Creutzfeldt W: Inhibition of glycogenolysis and glycogen phosphorylase by insulin and proinsulin in rat hepatocyte cultures. Diabetes 1987, 36(5):551-555.

36. Schudt C: Regulation of glycogen synthesis in rat-hepatocyte cultures by glucose, insulin and glucocorticoids. Eur J Biochem 1979, 97(1):155-160.

37. Veech RL, Raijman L, Dalziel K, Krebs HA: Disequilibrium in the triose phosphate isomerase system in rat liver. Biochem J 1969, 115(4):837-842.

38. Ikehara Y, Endo H, Okada Y: The identity of the aldolases isolated from rat muscle and primary hepatoma. Archives of biochemistry and biophysics 1970, 136(2):491-497.

39. Malay AD, Procious SL, Tolan DR: The temperature dependence of activity and structure for the most prevalent mutant aldolase B associated with hereditary fructose intolerance. Archives of biochemistry and biophysics 2002, 408(2):295-304.

40. Schuster R, Holzhutter HG: Use of mathematical models for predicting the metabolic effect of large-scale enzyme activity alterations. Application to enzyme deficiencies of red blood cells. Eur J Biochem 1995, 229(2):403-418.

41. Rider CC, Taylor CB: Enolase isoenzymes in rat tissues. Electrophoretic, chromatographic, immunological and kinetic properties. Biochimica et biophysica acta 1974, 365(1):285-300.

42. Meek DW, Nimmo HG: Effects of phosphorylation on the kinetic properties of rat liver fructose-1,6-bisphosphatase. Biochem J 1984, 222(1):125-130.

43. Cori CF, Velick SF, Cori GT: The combination of diphosphopyridine nucleotide with glyceraldehyde phosphate dehydrogenase. Biochimica et biophysica acta 1950, 4(1-3):160-169.

44. Ryzlak MT, Pietruszko R: Heterogeneity of Glyceraldehyde-3-Phosphate Dehydrogenase from Human-Brain. Biochimica et biophysica acta 1988, 954(3):309-324.

45. Smith CM, Velick SF: The glyceraldehyde 3-phosphate dehydrogenases of liver and muscle. Cooperative interactions and conditions for functional reversibility. The Journal of biological chemistry 1972, 247(1):273-284.

46. Bontemps F, Hue L, Hers HG: Phosphorylation of glucose in isolated rat hepatocytes. Sigmoidal kinetics explained by the activity of glucokinase alone. Biochem J 1978, 174(2):603-611.

47. Storer AC, Cornishbowden A: Kinetics of Rat-Liver Glucokinase - Cooperative Interactions with Glucose at Physiologically Significant Concentrations. Biochemical Journal 1976, 159(1):7-14.

48. Van Schaftingen E: A protein from rat liver confers to glucokinase the property of being antagonistically regulated by fructose 6-phosphate and fructose 1-phosphate. Eur J Biochem 1989, 179(1):179-184.

49. Agius L, Peak M: Intracellular binding of glucokinase in hepatocytes and translocation by glucose, fructose and insulin. Biochem J 1993, 296 ( Pt 3):785-796.

50. Igarashi Y, Kato S, Tada K: Kinetic properties of the glucose-6-phosphate transport system in rat hepatic microsomal membranes. J Inherit Metab Dis 1985, 8(3):153-154.

51. St-Denis JF, Berteloot A, Vidal H, Annabi B, van de Werve G: Glucose transport and glucose 6-phosphate hydrolysis in intact rat liver microsomes. The Journal of biological chemistry 1995, 270(36):21092-21097.

52. Ciaraldi TP, Horuk R, Matthaei S: Biochemical and Functional-Characterization of the Rat-Liver Glucose-Transport System - Comparisons with the Adipocyte Glucose-Transport System. Biochemical Journal 1986, 240(1):115-123.

53. Stalmans W, Gevers G: The catalytic activity of phosphorylase b in the liver. With a note on the assay in the glycogenolytic direction. Biochem J 1981, 200(2):327-336.

54. Tan AW, Nuttall FQ: Characteristics of the dephosphorylated form of phosphorylase purified from rat liver and measurement of its activity in crude liver preparations. Biochimica et biophysica acta 1975, 410(1):45-60.

55. Maddaiah VT, Madsen NB: Kinetics of purified liver phosphorylase. The Journal of biological chemistry 1966, 241(17):3873-3881.

56. Arion WJ, Nordlie RC: Liver Microsomal Glucose 6-Phosphatase, Inorganic Pyrophosphatase, and Pyrophosphate-Glucose Phosphotransferase. Ii. Kinetic Studies. The Journal of biological chemistry 1964, 239:2752-2757.

57. Kelmer-Bracht AM, Santos CP, Ishii-Iwamoto EL, Broetto-Biazon AC, Bracht A: Kinetic properties of the glucose 6-phosphatase of the liver from arthritic rats. Biochimica et biophysica acta 2003, 1638(1):50-56.

58. Tewari YB, Steckler DK, Goldberg RN: Thermodynamics of isomerization reactions involving sugar phosphates. The Journal of biological chemistry 1988, 263(8):3664-3669.

59. Zalitis J, Oliver IT: Inhibition of Glucose Phosphate Isomerase by Metabolic Intermediates of Fructose. Biochemical Journal 1967, 102(3):753-&.

60. Kashiwaya Y, Sato K, Tsuchiya N, Thomas S, Fell DA, Veech RL, Passonneau JV: Control of Glucose-Utilization in Working Perfused Rat-Heart. Journal of Biological Chemistry 1994, 269(41):25502-25514.

61. Colowick SP, Sutherland EW: Polysaccharide synthesis from glucose by means purified enzymes. Journal of Biological Chemistry 1942, 144(2):423-437.

62. Westphal SA, Nuttall FQ: Comparative characterization of human and rat liver glycogen synthase. Archives of biochemistry and biophysics 1992, 292(2):479-486.

63. Edlund GL, Halestrap AP: The kinetics of transport of lactate and pyruvate into rat hepatocytes. Evidence for the presence of a specific carrier similar to that in erythrocytes. Biochem J 1988, 249(1):117-126.

64. Anderson SR, Florini JR, Vestling CS: Rat Liver Lactate Dehydrogenase. 3. Kinetics and Specificity. The Journal of biological chemistry 1964, 239:2991-2997.

65. Prabhakaram M, Singh SN: Effect of age on the crystalline rat liver lactate dehydrogenase. Arch Gerontol Geriatr 1986, 5(1):57-64.

66. Williamson DH, Lund P, Krebs HA: The redox state of free nicotinamide-adenine dinucleotide in the cytoplasm and mitochondria of rat liver. Biochem J 1967, 103(2):514-527.

67. Indiveri C, Capobianco L, Kramer R, Palmieri F: Kinetics of the Reconstituted Dicarboxylate Carrier from Rat-Liver Mitochondria. Biochimica et biophysica acta 1989, 977(2):187-193.

68. Raval DN, Wolfe RG: Malic Dehydrogenase .4. Ph Dependence of Kinetic Parameters. Biochemistry 1962, 1(6):1118-&.

69. Crow KE, Braggins TJ, Batt RD, Hardman MJ: Rat liver cytosolic malate dehydrogenase: purification, kinetic properties, role in control of free cytosolic NADH concentration. Analysis of control of ethanol metabolism using computer simulation. The Journal of biological chemistry 1982, 257(23):14217-14225.

70. Thorne CJR: Properties of Mitochondrial Malate Dehydrogenases. Biochimica et biophysica acta 1962, 59(3):624-&.

71. Gelpi JL, Dordal A, Montserrat J, Mazo A, Cortes A: Kinetic-Studies of the Regulation of Mitochondrial Malate-Dehydrogenase by Citrate. Biochemical Journal 1992, 283:289-297.

72. Lynn R, Guynn RW: Equilibrium constants under physiological conditions for the reactions of succinyl coenzyme A synthetase and the hydrolysis of succinyl coenzyme A to coenzyme A and succinate. The Journal of biological chemistry 1978, 253(8):2546-2553.

73. Kimura N, Shimada N: Membrane-Associated Nucleoside Diphosphate Kinase from Rat-Liver - Purification, Characterization, and Comparison with Cytosolic Enzyme. Journal of Biological Chemistry 1988, 263(10):4647-4653.

74. Fukuchi T, Shimada N, Hanai N, Ishikawa N, Watanabe K, Kimura N: Recombinant rat nucleoside diphosphate kinase isoforms (alpha and beta): purification, properties and application to immunological detection of native isoforms in rat tissues. Biochimica et biophysica acta 1994, 1205(1):113-122.

75. Wimhurst JM, Manchester KL: Some aspects of the kinetics of rat liver pyruvate carboxylase. Biochem J 1970, 120(1):79-93.

76. Wood HG, Davis JJ, Lochmuller H: The equilibria of reactions catalyzed by carboxytransphosphorylase, carboxykinase, and pyruvate carboxylase and the synthesis of phosphoendolpyruvate. The Journal of biological chemistry 1966, 241(23):5692-5704.

77. Wilson DF, Erecinska M, Schramm VL: Evaluation of the relationship between the intra- and extramitochondrial [ATP]/[ADP] ratios using phosphoenolpyruvate carboxykinase. The Journal of biological chemistry 1983, 258(17):10464-10473.

78. Titheradge MA, Picking RA, Haynes RC: Physiological Concentrations of 2-Oxoglutarate Regulate the Activity of Phosphoenolpyruvate Carboxykinase in Liver. Biochemical Journal 1992, 285:767-771.

79. Colombo G, Carlson GM, Lardy HA: Phosphoenolpyruvate Carboxykinase (Guanosine Triphosphate) from Rat-Liver Cytosol - Separation of Homogeneous Forms of Enzyme with High and Low Activity by Chromatography on Agarose-Hexane-Guanosine Triphosphate. Biochemistry 1978, 17(25):5321-5329.

80. Ballard FJ, Hanson RW: Phosphoenolpyruvate carboxykinase and pyruvate carboxylase in developing rat liver. Biochem J 1967, 104(3):866-871.

81. Jo JS, Ishihara N, Kikuchi G: Occurrence and Properties of 4 Forms of Phosphoenolpyruvate Carboxykinase in Chicken Liver. Archives of biochemistry and biophysics 1974, 160(1):246-254.

82. Johnson TA, Holyoak T: Increasing the Conformational Entropy of the Omega-Loop Lid Domain in Phosphoenolpyruvate Carboxykinase Impairs Catalysis and Decreases Catalytic Fidelity. Biochemistry 2010, 49(25):5176-5187.

83. Ballard FJ: Kinetic studies with cytosol and mitochondrial phosphoenolpyruvate carboxykinases. Biochem J 1970, 120(4):809-814.

84. Holyoak T, Nowak T: pH dependence of the reaction catalyzed by avian mitochondrial phosphoenolpyruvate carboxykinase. Biochemistry 2004, 43(22):7054-7065.

85. Palmieri F, Quagliar.E, Stipani I, Klingenb.M: Kinetic Study of Tricarboxylate Carrier in Rat-Liver Mitochondria. European Journal of Biochemistry 1972, 26(4):587-&.

86. Uyeda K, Furuya E, Luby LJ: The Effect of Natural and Synthetic D-Fructose 2,6-Bisphosphate on the Regulatory Kinetic-Properties of Liver and Muscle Phosphofructokinases. Journal of Biological Chemistry 1981, 256(16):8394-8399.

87. Van Schaftingen E, Jett MF, Hue L, Hers HG: Control of liver 6-phosphofructokinase by fructose 2,6-bisphosphate and other effectors. Proc Natl Acad Sci U S A 1981, 78(6):3483-3486.

88. Reinhart GD, Lardy HA: Rat liver phosphofructokinase: kinetic activity under near-physiological conditions. Biochemistry 1980, 19(7):1477-1484.

89. Sakakibara R, Kitajima S, Uyeda K: Differences in kinetic properties of phospho and dephospho forms of fructose-6-phosphate, 2-kinase and fructose 2,6-bisphosphatase. The Journal of biological chemistry 1984, 259(1):41-46.

90. Van Schaftingen E, Davies DR, Hers HG: Inactivation of phosphofructokinase 2 by cyclic AMP - dependent protein kinase. Biochem Biophys Res Commun 1981, 103(1):362-368.

91. Vanschaftingen E, Davies DR, Hers HG: Fructose-2,6-Bisphosphatase from Rat-Liver. European Journal of Biochemistry 1982, 124(1):143-149.

92. Cornell NW, Leadbetter M, Veech RL: Effects of free magnesium concentration and ionic strength on equilibrium constants for the glyceraldehyde phosphate dehydrogenase and phosphoglycerate kinase reactions. The Journal of biological chemistry 1979, 254(14):6522-6527.

93. Krietsch WK, Bucher T: 3-phosphoglycerate kinase from rabbit sceletal muscle and yeast. Eur J Biochem 1970, 17(3):568-580.

94. Fritz PJ, White EL: 3-Phosphoglycerate kinase from rat tissues. Further characterization and developmental studies. Biochemistry 1974, 13(3):444-449.

95. Clarke JB, Birch M, Britton HG: The equilibrium constant of the phosphoglyceromutase reaction. Biochem J 1974, 139(3):491-497.

96. Fundele R, Krietsch WK: Purification and properties of the phosphoglycerate mutase isozymes from the mouse. Comp Biochem Physiol B 1985, 81(4):965-968.

97. Middleton MC, Walker DG: Comparison of the properties of two forms of pyruvate kinase in rat liver and determination of their separate activities during development. Biochem J 1972, 127(4):721-731.

98. van Berkel TJ, Kruijt JK, Koster JF: Hormone-induced changes in pyruvate kinase. Effects of glucagon and starvation. Eur J Biochem 1977, 81(3):423-432.

99. Walker PR, Potter VR, Becker JE, Bonney RJ: Pyruvate-Kinase, Hexokinase, and Aldolase Isoenzymes in Rat-Liver Cells in Culture. In Vitro Cell Dev B 1972, 8(2):107-&.

100. Titheradge MA, Coore HG: Mitochondrial Pyruvate Carrier, Its Exchange Properties and Its Regulation by Glucagon. FEBS letters 1976, 63(1):45-50.

101. Halestrap AP: The mitochondrial pyruvate carrier. Kinetics and specificity for substrates and inhibitors. Biochem J 1975, 148(1):85-96.

102. Lee EW, Barriso JA, Pepe M, Snyder R: Purification and Properties of Liver Triose Phosphate Isomerase. Biochimica et biophysica acta 1971, 242(1):261-&.

103. Turnquis.Rl, Gillett TA, Hansen RG: Uridine-Diphosphate Glucose Pyrophosphorylase - Crystallization and Properties of Enzyme from Rabbit Liver and Species Comparisons. Journal of Biological Chemistry 1974, 249(23):7695-7700.
