# Supplementary material for: A multiscale modelling approach to assess the impact of metabolic zonation and microperfusion on the hepatic carbohydrate metabolism
Source: PLoS Comput Biol. 2018 Feb 15;14(2):e1006005. doi: 10.1371/journal.pcbi.1006005 (PMC5841820; doi:10.1371/journal.pcbi.1006005)
Supplement: S2 Supplement — (DOCX) [file pcbi.1006005.s002.docx]

Supplement S2: sinusoidal blood flow model:

Fig 2: The sinusoidal blood flow model contains the blood vessel, the adjacent space of Disse and the surrounding hepatocyte cell layer (A)

The n individual hepatocytes aligned along the sinusoid are assumed to have homogenous metabolite concentration the vascular bed and the space of Disse are divided into n compartments assumed to be homogenous in metabolite and hormone concentration as well. Concentration changes for the different substances in the vascular bed and the space of Disse are given by the elementary processes considered in this model:

1. Directed lateral transport through substance flow from the periportal to pericentral region within the vascular bed
2. Lateral diffusion of substances along the vascular bed
3. Directional transversal substance exchange between the vascular bed and the space of Disse
4. Diffusional transversal substrate exchange between the blood vessel and the space of Disse
5. Directional lateral transport in the space of disse
6. Lateral diffusion of substances within the space of Disse
7. Substance exchange by active transport between the space of Disse and the hepatocytes

Lateral blood flow in the vessel compartment is described by Hagen-Poisson law in a cylinder. Water flow in the space of Disse is described by Hagen-Poisson law in a hollow cylinder. Exchange of water between the vessel and the space of Disse is driven by hydrostatic and oncotic pressure difference between the blood vessel and the space of disse. The dynamic substances in the liver sinusoid considered in our model are the metabolic active substances glucose and lactate, the hormones insulin and glucagon and the marker substances consisting of red blood cells, albumin and water.

| Symbol | Meaning | Model value | Experimental value / motivation | Citation |
| --- | --- | --- | --- | --- |
| Vol_cell_ | Hepatocyte volume | 3350 µm³ | ~5000 µm^3^ ; ~3000 µm^3^ | [[1](#_ENREF_1)]; [[2](#_ENREF_2)] |
| $r_{cell}$ | Radius hepatocyte | 7.5 µm |  |  |
| $l_{bv}$ | Segment length blood vessel | 15 µm | 2 $r_{cell}$ |  |
| $l_{disse}$ | Segment length space of disse | 15 µm | $l_{bv}$ |  |
| $r_{bv}$ | Blood vessel radius | 6.3±0.22 µm | 6.3±0.22 µm | [[3](#_ENREF_3)] |
| $A_{bv}$ | Blood vessel segment cross section area | 124.6 µm² | $\pi r_{bv}^{2}$ |  |
| Vol_blood_ | Blood vessel volume |  | $l_{bv} A_{bv}$ |  |
| $r_{disse}$ | Radius space of Disse | 1.28 ±0.53 µm (!min 0.1 µm) | 1.28 ±0.53 µm | [[4](#_ENREF_4)] |
| $A_{disse}$ | Disse segment cross section area | 55.8 µm² | $\pi\left( r_{bv}+r_{disse} \right)^{2}-A_{bv}$ |  |
| Vol_disse_ | Disse volume | 837 µm³ | $l_{disse} A_{disse}$ |  |
| $l_{trans}$ | Midpoint distance blood vessel/space of disse | 6.94 µm | $r_{sinusoid}+\frac{r_{disse}}{2}$ |  |
| $A_{trans}$ | Blood vessel/space of Disse segment contact area | 593 µm² | $2 {\pi l}_{bv} r_{bv}$ |  |
| $r_{sinusoid}$ | Radius of blood vessel and Disse combined | 7.58 µm | $r_{sinusoid}+r_{disse}$ |  |
| Vol_ER_ | Endoplasmatic volume | 3375 µm³ |  |  |
| Vol_mito_ | Mitochondrial volume | 562.5 µm³ |  |  |
| n | Number of hepatocytes along sinusoid | 20-25 | 20-25 | [[5](#_ENREF_5)] |
| $Perm$ | Permeability/degree of fenestration | 6%-8% | 6%-8% | [[6](#_ENREF_6)] |
| $r_{fen}$ | Fenestre radius | 70 nm | 70 nm | [[7](#_ENREF_7)] |
| $\sigma$ | Osmotic reflection coefficient | 1 | 1 | [[8](#_ENREF_8)] |
| $vis_{blood}$ | Blood viscosity | 0.004 Pa s | 0.003-0.004 Pa s | [[9](#_ENREF_9)] |
| $vis_{disse}$ | Lymph viscosity | 0.0018 Pa s | 0.0018 ± 0.00007 Pa s | [[10](#_ENREF_10)] |
| $p_{portal}$ | Portal (hydrostatic) blood pressure | 9.5 mmHg | 7-12 mmHg | [[11](#_ENREF_11)] |
| $\Delta HVPG$ | hepatic venous pressure gradient | 2±0.5 mmHg | 1-4 mmHg | [[11](#_ENREF_11)] |
| $p_{central}$ | Central (hydrostatic) blood pressure | 7.5 mmHg | $p_{portal}-\Delta HVPG$ |  |
| $p_{lymph}^{0}$ | Portal (hydrostatic) lymph pressure | 0 mmHg |  |  |
| $p_{lymph}^{n+1}$ | Central (hydrostatic) lymph pressure | 0 mmHg |  |  |
| $p_{onc-bv}^{i}$ | Oncotic pressure blood vessel | 23 mmHg | $\frac{p_{onc-bv}^{i}}{p_{onc-disse}^{i}}=0.95;$  $p_{onc-bv}^{i}-p_{onc-disse}^{i}=1-2mmHg$ | [[8](#_ENREF_8)] |
| $p_{onc-disse}^{i}$ | Oncotic pressure Disse | 21 mmHg | $\frac{p_{onc-bv}^{i}}{p_{onc-disse}^{i}}=0.95;$  $p_{onc-bv}^{i}-p_{onc-disse}^{i}=1-2mmHg$ | [[8](#_ENREF_8)] |
| $K_{H_{2}O}$ | Bulk modulus water | 1.564e7 mmHg |  |  |
| $D_{blood}^{rbc}$ | Plasma red blood cell diffusion coefficient | 1e-13 m²/s | 1e-13 m²/s | [[12](#_ENREF_12)] |
| $D_{blood}^{glc}$ | Plasma glucose diffusion coefficient | 6.7e-10 m^2^/s | 6.7e-10 m^2^/s | [[13](#_ENREF_13)] |
| $D_{blood}^{lac}$ | Plasma lactate diffusion coefficient | 1e-9 m²/s | 1e-9 m²/s | [[14](#_ENREF_14)] |
| $D_{blood}^{alb}$ | Plasma albumin diffusion coefficient | 6.8e-11 m²/s | 6.8e-11 m²/s | [[15](#_ENREF_15)] |
| $D_{blood}^{h_{2}o}$ | Plasma water diffusion coefficient | 3e-9 m²/s | 3e-9 m²/s | [[16](#_ENREF_16)] |
| $D_{blood}^{insulin}$ | Plasma insulin diffusion coefficient | 1.6e-10 m²/s | 1.6e-10 m²/s | [[17](#_ENREF_17)] |
| $D_{blood}^{glucagon}$ | Plasma glucagon diffusion coefficient | 1.6e-10 m²/s | like insulin |  |
| $Dif_{lymph}/Dif_{h20}$ | Relative lymph diffusion coefficient | 0.63 | 0.63 | [[10](#_ENREF_10)] |
| $D_{disse}^{glc}$ | Disse glucose diffusion coefficient | 4.22e-10 m²/s | $Dif_{lymph}/Dif_{h20}$ |  |
| $D_{disse}^{lac}$ | Disse lactate diffusion coefficient | 6.3e-10 m²/s | $Dif_{lymph}/Dif_{h20}$ |  |
| $D_{disse}^{alb}$ | Disse albumin diffusion coefficient | 4.28e-11 m²/s | $Dif_{lymph}/Dif_{h20}$ |  |
| $D_{disse}^{h_{2}o}$ | Disse water diffusion coefficient | 1.89e-9 m²/s | $Dif_{lymph}/Dif_{h20}$ |  |
| $D_{disse}^{insulin}$ | Disse insulin diffusion coefficient | 1.01e-10 m²/s | $Dif_{lymph}/Dif_{h20}$ |  |
| $D_{disse}^{glucagon}$ | Disse glucagon diffusion coefficient | 1.01e-10 m²/s | $Dif_{lymph}/Dif_{h20}$ |  |

***Blood vessel:***

Blood flow velocity /volume

$$v_{bloodflow-in}^{i}=\frac{r_{bv}^{2}}{8\cdot vis_{blood}\cdot l_{bv}}\cdot\Delta_{l}p_{bv}^{i}$$

$$\Delta_{l}p_{bv}^{i}=\left( p_{bv}^{i-1}-p_{bv}^{i} \right)$$

$$p_{bv}^{0}=p_{portal}$$

$$v_{Volflow_{in}-blood}^{i}=v_{bloodflow-in}^{i}\cdot A_{bv}$$

$$v_{bloodflow-out}^{i}=\frac{r_{bv}^{2}}{8\cdot vis_{blood}\cdot l_{bv}}\cdot\Delta_{r}p_{bv}^{i}$$

$$\Delta_{r}p_{bv}^{i}=\left( p_{bv}^{i}-p_{bv}^{i+1} \right)$$

$$p_{bv}^{n+1}=p_{central}$$

$$v_{Volflow_{out}-blood}^{i}=v_{bloodflow-out}^{i}\cdot A_{bv}$$

***Space of Disse:***

$$v_{lymphflow-in}^{i}=\frac{r_{sinusoid}^{2} +r_{bv}^{2}-\frac{\left( r_{sinusoid}^{2}-r_{bv}^{2} \right)}{ln\left( \frac{r_{sinusoid}}{r_{bv}} \right)}}{4\cdot vis_{blood}\cdot l_{bv}}\cdot\Delta_{l}p_{lymph}^{i}$$

$$\Delta_{l}p_{lymph}^{i}=\left( p_{lymph}^{i-1}-p_{lymph}^{i} \right)$$

$$v_{Volflow_{in}-disse}^{i}= \pi\cdot\left[ \left( r_{bv}^{i}+r_{sinusoid}^{i} \right)^{2}-{r_{bv}^{i}}^{2} \right]\cdot v_{lymphflow-in}^{i}$$

$$v_{lymphflow-out}^{i}=\frac{r_{sinusoid}^{2} +r_{bv}^{2}-\frac{\left( r_{sinusoid}^{2}-r_{bv}^{2} \right)}{ln\left( \frac{r_{sinusoid}}{r_{bv}} \right)}}{4\cdot vis_{blood}\cdot l_{bv}}\cdot\Delta_{r}p_{lymph}^{i}$$

$$\Delta_{r}p_{lymph}^{i}=\left( p_{lymph}^{i}-p_{lymph}^{i+1} \right)$$

$$v_{Volflow_{out}-disse}^{i}=\pi\cdot\left[ \left( r_{bv}^{i}+r_{sinusoid}^{i} \right)^{2}-{r_{bv}^{i}}^{2} \right]\cdot v_{lymphflow-out}^{i}$$

***Transversal flow:***

$$v_{transversal}^{i}=\frac{r_{fen}^{2}}{4\cdot vis_{blood}\cdot l_{trans}}\cdot\left( \Delta p_{hydrostatic}^{i}-\sigma\cdot\Delta p_{oncotic}^{i} \right)$$

$$\Delta p_{hydrostatic}^{i}=\left( p_{bv}^{i}-p_{lymph}^{i} \right)$$

$$\Delta p_{oncotic}^{i}=\left( p_{onc-bv}^{i}-p_{onc-lymph}^{i} \right)$$

$$v_{Volflow-transversal}^{i}=Perm\cdot{A_{blood/disse}^{i}\cdot v}_{transversal}^{i}$$

***Metabolite flow in blood vessel***:

$$v_{X_{blood}-directional}^{i}=v_{Volflow_{in}-blood}^{i}\cdot\left[ X_{blood}^{i-1} \right]-v_{Volflow_{out}-blood}^{i}\cdot\left[ X_{blood}^{i} \right]$$

***Metabolite flow in space of Disse:***

$$v_{X_{disse}-directional}^{i}=v_{Volflow_{in}-disse}^{i}\cdot\left[ X_{disse}^{i-1} \right]-v_{Volflow_{out}-disse}^{i}\cdot\left[ X_{disse}^{i} \right]$$

***Transversal metabolite flow:***

$$v_{transversal}^{i}=v_{Volflow-transversal}^{i}\cdot\left[ X_{blood}^{i} \right] if v_{Volflow-transversal}^{i}>0$$

$$v_{transversal}^{i}=v_{Volflow-transversal}^{i}\cdot\left[ X_{lymph}^{i} \right] if v_{Volflow-transversal}^{i}<0$$

***Diffusive metabolite flow in blood vessel:***

$$v_{X_{blood}-diffusion}^{i}=\frac{D_{blood}^{X}\cdot A_{bv}}{l_{bv}} \Delta\left[ X_{blood}^{i} \right]$$

$$D_{blood}^{X}= \in\left\{ D_{blood}^{glc}, D_{blood}^{lac}, D_{blood}^{alb}, D_{blood}^{h_{2}o} \right\}$$

$$\Delta\left[ X_{blood}^{i} \right]= \left[ X_{blood}^{i-1} \right]-2\cdot\left[ X_{blood}^{i} \right]+\left[ X_{blood}^{i+1} \right]$$

***Diffusive metabolite flow in space of Disse:***

$$v_{X_{disse}-diffusion}^{i}=\frac{D_{disse}^{X}\cdot A_{disse}}{l_{disse}} \Delta\left[ X_{disse}^{i} \right]$$

$$D_{disse}^{X}=\in\left\{ D_{disse}^{glc}, D_{disse}^{lac}, D_{disse}^{alb}, D_{disse}^{h_{2}o} \right\}$$

$$\Delta\left[ X_{disse}^{i} \right]= \left[ X_{disse}^{i-1} \right]-2\cdot\left[ X_{disse}^{i} \right]+\left[ X_{disse}^{i+1} \right]$$

***Diffusive transversal metabolite flow:***

$$v_{X_{trans}-diffusion}^{i}=\frac{D_{trans}^{X}\cdot A_{trans}}{l_{trans}}\cdot Perm\cdot\Delta\left[ X_{trans}^{i} \right]$$

$$D_{trans}^{X}=$$

$$\Delta\left[ X_{trans}^{i} \right]= \left[ X_{blood}^{i} \right]-\left[ X_{disse}^{i} \right]$$

***Stoichiometric matrix:***

$$\frac{d\left[ glc_{ext}^{i} \right]}{dt}=\frac{v_{glc_{blood}-directional}^{i}}{Vol_{blood}^{i}} + \frac{v_{glc_{blood}-diffusion}^{i}}{Vol_{blood}^{i}} - \frac{v_{glc_{trans}-directional}^{i}}{Vol_{blood}^{i}} - \frac{v_{glc_{trans}-diffusion}^{i}}{Vol_{blood}^{i}}$$

$$\frac{d\left[ glc_{dis}^{i} \right]}{dt}=\frac{v_{glc_{dis}-directional}^{i}}{Vol_{disse}^{i}} + \frac{v_{glc_{dis}-diffusion}^{i}}{Vol_{disse}^{i}}+ \frac{v_{glc_{trans}-directional}^{i}}{Vol_{disse}^{i}}+ \frac{v_{glc_{trans}-diffusion}^{i}}{Vol_{disse}^{i}}$$

$$\frac{d\left[ lac_{ext}^{i} \right]}{dt}=\frac{v_{l{ac}_{blood}-directional}^{i}}{Vol_{blood}^{i}} + \frac{v_{lac_{blood}-diffusion}^{i}}{Vol_{blood}^{i}} - \frac{v_{lac_{trans}-directional}^{i}}{Vol_{blood}^{i}} - \frac{v_{lac_{trans}-diffusion}^{i}}{Vol_{blood}^{i}}$$

$$\frac{d\left[ lac_{dis}^{i} \right]}{dt}=\frac{v_{lac_{dis}-directional}^{i}}{Vol_{disse}^{i}} + \frac{v_{lac_{dis}-diffusion}^{i}}{Vol_{disse}^{i}}+ \frac{v_{lac_{trans}-directional}^{i}}{Vol_{disse}^{i}}+ \frac{v_{lac_{trans}-diffusion}^{i}}{Vol_{disse}^{i}}$$

$$\frac{d\left[ alb_{blood-label}^{i} \right]}{dt}=\frac{v_{{alb}_{blood-label}-directional}^{i}}{Vol_{blood}^{i}}+ \frac{v_{{alb}_{blood-label}-diffusion}^{i}}{Vol_{blood}^{i}}- \frac{v_{{alb}_{trans -label}-directional}^{i}}{Vol_{blood}^{i}}- \frac{v_{{alb}_{trans-label}-diffusion}^{i}}{Vol_{blood}^{i}}$$

$$\frac{d\left[ alb_{dis-label}^{i} \right]}{dt}=\frac{v_{{alb}_{disse-label}-directional}^{i}}{Vol_{disse}^{i}}+ \frac{v_{{alb}_{disse-label}-diffusion}^{i}}{Vol_{disse}^{i}}+ \frac{v_{{alb}_{trans -label}-directional}^{i}}{Vol_{disse}^{i}}+ \frac{v_{{alb}_{trans-label}-diffusion}^{i}}{Vol_{disse}^{i}}$$

$$\frac{d\left[ h_{2}o_{blood-label}^{i} \right]}{dt}=\frac{v_{h_{2}o_{blood-label}-directional}^{i}}{Vol_{blood}^{i}}+ \frac{v_{h_{2}o_{blood-label}-diffusion}^{i}}{Vol_{blood}^{i}}- \frac{v_{h_{2}o_{trans -label}-directional}^{i}}{Vol_{blood}^{i}}- \frac{v_{h_{2}o_{trans-label}-diffusion}^{i}}{Vol_{blood}^{i}}$$

$$\frac{d\left[ h_{2}o_{dis-label}^{i} \right]}{dt}=\frac{v_{h_{2}o_{disse-label}-directional}^{i}}{Vol_{disse}^{i}}+ \frac{v_{h_{2}o_{disse-label}-diffusion}^{i}}{Vol_{disse}^{i}}+ \frac{v_{h_{2}o_{blood/disse -label}-directional}^{i}}{Vol_{disse}^{i}}+ \frac{v_{h_{2}o_{blood/disse-label}-diffusion}^{i}}{Vol_{disse}^{i}}$$

$$\frac{dp_{bv}^{i}}{dt}=K_{H_{2}O}\cdot\left( v_{Volflow_{in}-blood}^{i}-v_{Volflow_{out}-blood}^{i}-v_{Volflow-transversal}^{i} \right)$$

$$\frac{dp_{disse}^{i}}{dt}=K_{H_{2}O}\cdot\left( v_{Volflow_{in}-disse}^{i}-v_{Volflow_{out}-disse}^{i}+v_{Volflow-transversal}^{i} \right)$$

1. Staubli, W., R. Hess, and E.R. Weibel, *Correlated morphometric and biochemical studies on the liver cell. II. Effects of phenobarbital on rat hepatocytes.* J Cell Biol, 1969. **42**(1): p. 92-112.

2. Wisniewski, J.R., et al., *In-depth quantitative analysis and comparison of the human hepatocyte and hepatoma cell line HepG2 proteomes.* Journal of Proteomics, 2016. **136**: p. 234-247.

3. Koo, A. and I.Y. Liang, *Microvascular filling pattern in rat liver sinusoids during vagal stimulation.* J Physiol, 1979. **295**: p. 191-9.

4. Hung, D.Y., et al., *Quantitative evaluation of altered hepatic spaces and membrane transport in fibrotic rat liver.* Hepatology, 2002. **36**(5): p. 1180-9.

5. Fausto, N. and J.S. Campbell, *The role of hepatocytes and oval cells in liver regeneration and repopulation.* Mechanisms of Development, 2003. **120**(1): p. 117-130.

6. Wisse, E., et al., *Scanning electron microscope observations on the structure of portal veins, sinusoids and central veins in rat liver.* Scan Electron Microsc, 1983(Pt 3): p. 1441-52.

7. Wisse, E., et al., *The size of endothelial fenestrae in human liver sinusoids: implications for hepatocyte-directed gene transfer.* Gene Therapy, 2008. **15**(17): p. 1193-1199.

8. Laine, G.A., et al., *Transsinusoidal fluid dynamics in canine liver during venous hypertension.* Circ Res, 1979. **45**(3): p. 317-23.

9. Elert, G. *Viscosity. The Physics Hypertextbook.* . 2017.

10. Bouta, E.M., et al., *In vivo quantification of lymph viscosity and pressure in lymphatic vessels and draining lymph nodes of arthritic joints in mice.* Journal of Physiology-London, 2014. **592**(6): p. 1213-1223.

11. Armonis, A., D. Patch, and A. Burroughs, *Hepatic venous pressure measurement: an old test as a new prognostic marker in cirrhosis?* Hepatology, 1997. **25**(1): p. 245-8.

12. Higgins, J.M., et al., *Statistical Dynamics of Flowing Red Blood Cells by Morphological Image Processing.* Plos Computational Biology, 2009. **5**(2).

13. Longsworth, L.G., *Diffusion Measurements, at 25-Degrees, of Aqueous Solutions of Amino Acids, Peptides and Sugars.* Journal of the American Chemical Society, 1953. **75**(22): p. 5705-5709.

14. Ribeiro, A.C.F., et al., *Binary diffusion coefficients for aqueous solutions of lactic acid.* Journal of Solution Chemistry, 2005. **34**(9): p. 1009-1016.

15. Wakeham, W.A., N.H. Salpadoru, and C.G. Caro, *Diffusion-Coefficients for Protein Molecules in Blood-Serum.* Atherosclerosis, 1976. **25**(2-3): p. 225-235.

16. Holz, M., S.R. Heil, and A. Sacco, *Temperature-dependent self-diffusion coefficients of water and six selected molecular liquids for calibration in accurate H-1 NMR PFG measurements.* Physical Chemistry Chemical Physics, 2000. **2**(20): p. 4740-4742.

17. Jensen, S.S., et al., *Insulin diffusion and self-association characterized by real-time UV imaging and Taylor dispersion analysis.* Journal of Pharmaceutical and Biomedical Analysis, 2014. **92**: p. 203-210.
