# Supplementary material for: A multiscale modelling approach to assess the impact of metabolic zonation and microperfusion on the hepatic carbohydrate metabolism
Source: PLoS Comput Biol. 2018 Feb 15;14(2):e1006005. doi: 10.1371/journal.pcbi.1006005 (PMC5841820; doi:10.1371/journal.pcbi.1006005)
Supplement: S3 Supplement — (DOCX) [file pcbi.1006005.s003.docx]

## Signaling transfer functions ([Bulik et al, 2016](#_ENREF_2))

**Glucose-Hormone Transfer function (GHT)**

This function relates the plasma level of glucose to the plasma levels of insulin (Ins) and glucagon.

$\mathrm{Ins}_{\mathrm{disse}_{0}}=2\cdot1.55 nM\cdot\frac{\left( \mathrm{Gl}c_{\mathrm{ext}} \right)^{5.7}}{\left( \mathrm{Gl}c_{\mathrm{ext}} \right)^{5.7}+\left( 7.7 mM \right)^{5.7}}$

$\mathrm{Glucagon}_{\mathrm{disse}_{0}}=2\cdot\left( 0.253 nM\cdot\left( 1-\frac{\left( \mathrm{Gl}c_{\mathrm{ext}} \right)^{5.65}}{\left( \mathrm{Gl}c_{\mathrm{ext}} \right)^{5.65}+\left( 4.7 mM \right)^{5.65}} \right)+0.02 nM \right)$

**Hormone-Phosphorylation Transfer function**

This function relates the plasma level of of insulin (Ins) and glucagon to the phosphorylation state of hormone sensitive enzymes. γ represents the fraction of enzyme protein in the phosphorylated state.

$\gamma=\frac{1}{2}*\left( 1-\frac{Ins^{1.75}}{Ins^{1.75}+\left( 0.70 nM \right)^{1.75}}+\frac{Glucagon^{3.6}}{Glucagon^{3.6}+\left( 0.08 nM \right)^{3.6}} \right)$

## Sinusoidal clearance of insulin and glucagon

$v_{clear}^{Glucagon}=v_{max}^{Glucagon_{clear}} \cdot\frac{Glucagon_{disse}}{Glucagon_{disse}+k_{m}^{Glucagon_{disse}}}$

$v_{max}^{Glucagon_{clear}}=288 \mu M\cdot g^{-1}\cdot h^{-1}$ ([Balks & Jungermann, 1984](#_ENREF_1))

$k_{m}^{Glucagon_{disse}}=80 pM$

$v_{clear}^{Ins}=v_{max}^{Ins_{clear}} \cdot\frac{{Ins}_{disse}}{{Ins}_{disse}+k_{m}^{{Ins}_{disse}}}$

$v_{max}^{{Ins}_{clear}}=1620 \mu M\cdot g^{-1}\cdot h^{-1}$ ([Balks & Jungermann, 1984](#_ENREF_1))

$k_{m}^{{Ins}_{disse}}=210 pM$

## Addition to stoichiometric matrix:

$$\frac{d}{dt}Ins_{disse}= {-v}_{clear}^{Ins}$$

$$\frac{d}{dt}{Glucagon}_{disse}= {-v}_{clear}^{Glucagon}$$

Balks HJ, Jungermann K (1984) Regulation of Peripheral Insulin Glucagon-Levels by Rat-Liver. *Eur J Biochem* **141:** 645-650

Bulik S, Holzhutter HG, Berndt N (2016) The relative importance of kinetic mechanisms and variable enzyme abundances for the regulation of hepatic glucose metabolism--insights from mathematical modeling. *BMC biology* **14:** 15
